# Probing the 3D architecture of the plant nucleus with microscopy approaches: challenges and solutions

**DOI:** 10.1080/19491034.2019.1644592

**Published:** 2019-07-30

**Authors:** Tao Dumur, Susan Duncan, Katja Graumann, Sophie Desset, Ricardo S Randall, Ortrun Mittelsten Scheid, Dimiter Prodanov, Christophe Tatout, Célia Baroux

**Affiliations:** a Gregor Mendel Institute (GMI) of Molecular Plant Biology, Austrian Academy of Sciences, Vienna Biocenter (VBC), Vienna, Austria; b Norwich Research Park, Earlham Institute, Norwich, UK; c Department of Biological and Medical Sciences, Oxford Brookes University, Oxford, UK; d GReD, Université Clermont Auvergne, CNRS, INSERM, Clermont–Ferrand, France; e Department of Plant and Microbial Biology, Zürich-Basel Plant Science Center, University of Zürich, Zürich, Switzerland; f Environment, Health and Safety, Neuroscience Research Flanders, Leuven, Belgium

**Keywords:** Three-dimensional microscopy imaging, image processing and analysis, tracking, plant nucleus, live imaging, whole-mount, nuclear organization

## Abstract

The eukaryotic cell nucleus is a central organelle whose architecture determines genome function at multiple levels. Deciphering nuclear organizing principles influencing cellular responses and identity is a timely challenge. Despite many similarities between plant and animal nuclei, plant nuclei present intriguing specificities. Complementary to molecular and biochemical approaches, 3D microscopy is indispensable for resolving nuclear architecture. However, novel solutions are required for capturing cell-specific, sub-nuclear and dynamic processes. We provide a pointer for utilising high-to-super-resolution microscopy and image processing to probe plant nuclear architecture in 3D at the best possible spatial and temporal resolution and at quantitative and cell-specific levels. High-end imaging and image-processing solutions allow the community now to transcend conventional practices and benefit from continuously improving approaches. These promise to deliver a comprehensive, 3D view of plant nuclear architecture and to capture spatial dynamics of the nuclear compartment in relation to cellular states and responses.

**Abbreviations:** 3D and 4D: Three and Four dimensional; AI: Artificial Intelligence; ant: antipodal nuclei (ant); CLSM: Confocal Laser Scanning Microscopy; CTs: Chromosome Territories; DL: Deep Learning; DLIm: Dynamic Live Imaging; ecn: egg nucleus; FACS: Fluorescence-Activated Cell Sorting; FISH: Fluorescent In Situ Hybridization; FP: Fluorescent Proteins (GFP, RFP, CFP, YFP, mCherry); FRAP: Fluorescence Recovery After Photobleaching; GPU: Graphics Processing Unit; KEEs: KNOT Engaged Elements; INTACT: Isolation of Nuclei TAgged in specific Cell Types; LADs: Lamin-Associated Domains; ML: Machine Learning; NA: Numerical Aperture; NADs: Nucleolar Associated Domains; PALM: Photo-Activated Localization Microscopy; Pixel: Picture element; pn: polar nuclei; PSF: Point Spread Function; RHF: Relative Heterochromatin Fraction; SIM: Structured Illumination Microscopy; SLIm: Static Live Imaging; SMC: Spore Mother Cell; SNR: Signal to Noise Ratio; SRM: Super-Resolution Microscopy; STED: STimulated Emission Depletion; STORM: STochastic Optical Reconstruction Microscopy; syn: synergid nuclei; TADs: Topologically Associating Domains; Voxel: Volumetric pixel

## Introduction

The cell’s nucleus is much more than a genetic container, it plays a fundamental role in coordinating transcription, replication, and repair and in integrating cellular parameters and environmental signals. Although plant and animal eukaryotes diverged two billion years ago, their nuclei share several organising principles that can be considered as the fundamental features of functional nuclear architecture. These include the formation of Chromosome Territories (CT) at interphase; the combinatorial principle and distribution of histone modifications and histone variants; the organisation of chromatin domains cytologically defined as eu- and heterochromatin (*e.g*. chromocenters and interspersed heterochromatic knobs); the formation of chromatin regions *in trans* within structured domains; the compartmentalisation of functional areas enriched in specific proteins and RNA called nuclear bodies; and the presence of a nucleo-cytoplasmic interface: the nuclear envelope with the nuclear pores (see reviews [1–5], Despite recent progress, knowledge of the organisation and dynamics of the plant nucleus remains sparse when compared to the animal field. One reason for this disparity comes from challenges specific to cytological approaches in plant tissues. Another reason is the difficulty to finance fundamental projects about the nuclear organisation – due to current priorities in plant science, funding programmes meet preferentially agronomy/ecology challenges. Yet, there is increasing recognition of the need to expand our knowledge of the nuclear organisation in other eukaryotic models, notably plants. Comparing nuclear organisation in plants and animal eukaryotes [2,6] offers the great promise to highlight evolutionary-relevant functional features relying on the nuclear organisation. In this effort, the INDEPTH consortium aims at elucidating the functional architecture of plant nuclei, particularly in relation to plant phenotypes relevant to both basic and applied research [7]. This initiative will foster focused investigations in the understudied area. It aims to provide valuable insights into the innovative evolutionary strategies evolved in the plant kingdom, to discover novel routes to understanding and eventually controlling the phenotypic plasticity of plant nuclei and to ultimately contribute solutions to ecologically and agriculturally relevant problems.

### Breaking the dogma of a unique nuclear organisation model – specificities of plant nuclei

The plant nucleus shows specific structural, compositional and functional features. It displays remarkable variation in nuclear shape, size, ploidy, in distribution and composition of nuclear domains, and in heterochromatin content and chromatin condensation. There is also a variable distribution of chromatin marks throughout the life cycle (*i.e*. upon developmental cues) and in response to the environment, such as during biotic and abiotic stresses (reviewed in [3,8–13].

One such plant-specific nuclear feature is the nucleoskeleton, a peripheral matrix functionally comparable to, but structurally different from, the lamina in animal cells, and much less well investigated. Putative protein components of the nucleoskeleton do not share any significant sequence homology with animal Lamin A/C and B. This raises intriguing questions regarding the evolution of the structure and function of the nuclear periphery in plant and animal cells [1,14]. Nevertheless, peripheral chromatin regions called Plant Lamina-Associated Domains (PLADs) were identified first as chromatin regions associated with NUP1/136, a component of the nuclear pore complex [15] and more recently with CRWN1, a component of the nucleoskeleton [16]. Like in animal cells, the nucleoskeleton of the plant nucleus may function to tether chromatin domains -as seen by ChIP and chromosome painting- that carry repressive features [15,16]. Peripheral localisation of these domains seems dependent upon non-CG methylation [16], a DNA modification unique to plants, hence representing plant-specific novelties in LAD regulation and function.

Another distinctive feature is the 3D organisation of plant genomes in terms of spatial interactions and associations. Recent reviews provide well-illustrated and documented comparisons highlighting similarities and peculiarities in the different genome organisation scale – from gene to megabase levels – corresponding to structural domains, A/B compartments and Topologically Associated Domains (TADs) [4,6]. One example that merits attention is the TAD: while being well-conserved features of metazoans [17], TADs are not robustly detected in Arabidopsis [5,18]. TADs have however been identified in other plant species with larger genomes that include more repeated sequences dispersed along the chromosomes [19,20]. This suggests the possibility that, although TADs have evolved in both plant and animal genomes, their role in genome function differs; notably plant TADs may not represent functional units of gene regulation as in animal cells [6] .

In animal cells, spatial gene re-positioning upon transcription – relative to nuclear bodies and CTs – and the clustering of gene loci into transcription factories are considered key organisational principles [21]. In plants, the situation is unclear. While gene relocation has been described for a subset of loci in Arabidopsis, there is currently no evidence that this represents a paradigm in plant cells, nor that transcription factories really occur [10,21–25]. It could, however, be that the establishment of unambiguous models is hampered by the heterogeneity of nuclear organization in plant tissues (unlike animals, plants cannot be cultured in homogenous cell lineages) and their plasticity in response to environmental stimuli. Thus, there is a clear need to transcend single-case studies and implement innovative approaches to probe the spatial organisation of the transcriptional compartment at the genome-wide level and to elucidate the functional relationships between transcribed loci and their spatial arrangement in the plant cell nucleus.

### How to probe the spatial arrangement of the plant nucleus?

A major advance over recent decades has been a paradigm shift from a static to a dynamic view of nuclear organisation. Understanding the functional relationship between plasticity and dynamic processes, both at the nuclear and at the organismal level and during development and physiological adaptation, is a major challenge [3,7,26]. However, combined methodologies need to be applied and further developed to probe the plant cell nucleus in its full complexity during cellular responses – ideally in a cell/tissue – specific manner. Cytological, molecular and biochemical approaches collectively yield insights into the spatial organisation of functional domains, chart chromatin complexes, histone modifications and variants along the genome and provide spatial statistical models of 3D organisation. A compendium of high-end protocols optimized by several laboratories was recently compiled for the community (*Plant Chromatin Dynamics: Methods and Protocols* [27]). In addition, the INDEPTH consortium now aims to share current state-of-the-art microscopy and imaging technologies (among other approaches) to elucidate functional principles of the plant nucleus [7].

In this commentary paper, we review the possibilities and challenges specifically offered by microscopy imaging and image processing to probe the 3D organisation of plant cell nuclei. We present a few case studies and discuss some general considerations for scientists in the field. We have focused on discussing the benefits and limitations of imaging fixed *versus* living tissue, dissected fragments or isolated nuclei *versus* whole-mount and fluorescent labelling of endogenous components *versus* artificial tagging of a protein-of-interest. Deciding on the best strategy is not an easy task and requires the consideration of many parameters. These include, but are not limited to, the biological tissue-of-interest, optical properties, the necessity to maintain tissue integrity and the capacity for generating transgenic lines expressing tagged proteins in species-of-interests. There is often no single solution that overcomes all constraints, but it is useful to consider several approaches collectively to gain information. This report aims to be a useful reference for plant cell biologists wishing to image nuclear processes and an aid for selecting the best imaging technique and image processing design to meet a variety of needs.

## Imaging the plant cell nucleus: in whole-mount or following isolation – different modalities

### Important considerations prior to imaging

#### Optical properties of plant tissues

The optical properties of plant tissues pose significant challenges to imaging of nuclei in whole-mount tissues or living cells. Pigments and diverse cellular compounds are notorious for generating autofluorescence since they absorb light at different wavelengths and possibly also fluoresce. This is true for chlorophyll pigments, alkaloids, flavonoids, nicotinamides and cell wall compounds for which subcellular localisation has been well documented [28–32]. Autofluorescence is particularly prominent in old and stressed tissue [29,33]. Of importance for imaging the plant cell nucleus, flavonoids can also accumulate in the nucleoplasm [33]. Thus imaging of fluorescent nuclear proteins and fluorescent dyes in fresh tissues or living cells can lead to complex emission patterns. This necessitates a careful selection of fluorophores with distinct and non-overlapping emission spectra to minimize the impact of any potential background emissions. Alternatively, spectral unmixing can be used to separate each contribution [34]. Furthermore, chloroplasts, mitochondria, starch granules and the cell wall are all light-scattering structures [35–37]. Collectively, background light absorbing, emitting and scattering factors reduce both excitation and signal detection efficiencies in whole-mount fresh tissues. This inevitably leads to loss of signal contrast, which translates to a reduction in effective resolution [38]. This particularly affects the sub-micrometric scale and thereby impedes resolution of closely-related and complex subnuclear structures. An alternative to imaging in fresh tissue consists in applying optical clearance methods on fixed tissue preparations. Imaging isolated nuclei can also circumvent these problems. The benefits and limitations of each approach are discussed in the following sections.

#### Checklist for a good imaging design

At the start of each imaging experiment, it is important to careful evaluate (i) the choice of fluorophore, (ii) sample preparation, (iii) the optical/microscopy setup and (iv) the image acquisition parameters. We refer to this collectively as *imaging design*. A good imaging design allows to resolve fine-scale details in the plant nucleus, as shown by several illustrations in this review. Conventional microscopy, accessible to the majority of plant cell biologists, allows to resolve, for instance, large chromatin domains; genomic regions; protein complexes forming speckles or located in nuclear bodies in the 200 nm range or below and detected by immunostaining, *in situ* hybridization or tagged fluorescent proteins.

Expedited routine practices tend towards a simplified imaging design at the detriment of signal quality and signal contrast, hence poor spatial resolution. General considerations of image resolution [38,39], and more generally of microscopy imaging of plant tissues, are published elsewhere. This reading material is strongly recommended prior to starting an experiment [37,40,41], to avoid classical pitfalls and to prepare well for image analysis and quantification [39,42,43]. More directly related to imaging the plant cell nucleus, we recently reviewed practical considerations for plant chromatin imaging, including a comparison of imaging instruments and their resolution [44]. The motivation for a good imaging design is to achieve the best-possible resolution within a constrained imaging procedure, including sample viability and accessibly, available fluorophores and imaging systems.

Imaging design should consider the following issues:

- *fluorophores*: the property of the fluorophore (FP, dye or conjugated antibody) is a primary consideration, and efforts should be made wherever possible to avoid low quantum efficiency and poor photostability (for live reporters, see reference [45] for a comparison of several FPs). In addition, specific fluorophores are required for super-resolution imaging instruments [46,47]. As mentioned, multiple fluorophores should offer well-separated spectral properties among each other and potential background fluorescence [34].
- *sample preparation* will greatly influence the Signal-to-Noise Ratio (SNR, which measures the amount of informative signal relative to undesired, background noise) and thus merits serious consideration. In addition to endogenous fluorescence and the scattering properties detailed above, the mounting of thick samples or the presence of air bubbles trapped in the preparation will reduce excitation efficiency and signal collection considerably due to low light penetration and light scattering at each interface. Mounting and lens immersion media should be matched with identical (or as similar as possible) refractive indices [35,48].
- *microscopy setup*. While appearing a basic recommendation, it is important to select an objective lens with suitable Numerical Aperture (NA), immersion medium, working distance and imaging depth and corrections for chromatic and axial aberrations.
- *image acquisition*. We do not discuss here manufacturer-specific recommendations, but recall that image resolution and the possibility to perform downstream quantification depend on signal contrast, signal quality and intensity range [38,39]. Hence resolving fine-scale nuclear structures requires a well-thought setup for signal acquisition. A conservative practice includes avoiding near-saturation intensity levels and averaging modes as these may artificially alter intensity distributions and negatively influence the SNR. Photon-counting modes with signal accumulation are normally preferable to the use of gain. Where available, new-generation sensitive detectors and fast imaging systems should be considered as means to minimize the risk of photobleaching. These technologies include resonance scanning modes and spinning disk or light-sheet imaging systems. The latter is the most expensive option of the three and is of limited interest for fine-scale nuclei analysis, as it offers reduced spatial resolution. Conversely, super resolution imaging approaches are strongly limited in imaging depth (see 1.4). Thus, the level of resolution required for each experiment should be carefully evaluated in view of the necessity to preserve tissue architecture.

#### Realistic resolution levels with current imaging practices

Spatial resolution referred to in imaging practices defines the minimal distance between two objects that allows for the distinction of each by means of signal contrast [38,39]. In conventional compound microscopes, optical resolution is determined primarily by the NA of the objective. However, in wide-field microscopy, the collection of out-of-focus, scattered light hinders the achievement of optimal resolution. This problem is solved by pinhole detectors in confocal imaging systems. Resolution is then influenced by the pinhole size (variable in conventional Confocal Laser Scanning Microscopy, CLSM, fixed in spinning disk systems), the detector and the image binning factor [38,39,49].

**Figure 1.**
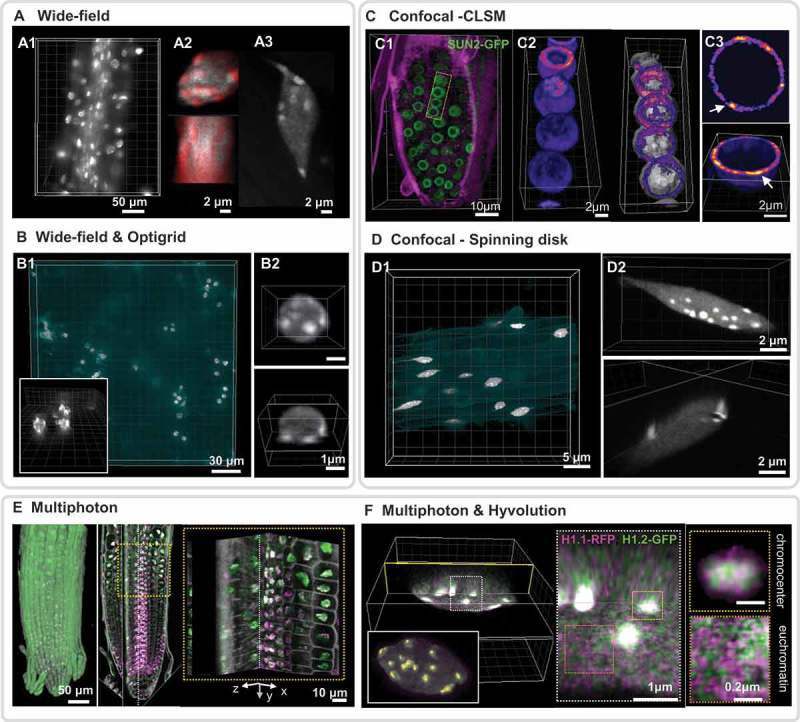
Imaging plant nuclei in live (fresh) whole-mount tissue. All images illustrate a nuclear staining in whole-mount with an overview of the tissue/organ (left) and close-up images (right panels) showing orthogonal sections or 3D rendering (powered by Imaris, Bitplane AG, CH). (a) Wide-field imaging (Leica DM6000) Fresh seedling root (Arabidopsis) stained in whole-mount with DAPI in 0.5xMS and 1% sucrose, imaged with an oil immersion objective (40x NA 1.3). Overview (max. projection (a1), detail of a single nucleus from the cortext zone (orthogonal sections, (a2), DAPI (grey) and H1.1-RFP (red), and detail of an epidermis nucleus (max.projection (a3), DAPI only. The image quality is suitable for quantification of the heterochromatin content. Image source CB. (b) Wide-field imaging (Leica DM6000) & Optigrid-based restoration. Fresh cotyledon (Arabidopsis) stained in whole mount with DAPI and imaged in oil-immersion objective (63x NA 1.4). Overview (max. projection (b1). grey, DAPI. The background fluorescence has been pseudocolored in cyan) and zoom on part of the image (insert). Detail of a single nucleus cropped from the large view (b2). Image source CT & SD. (c) Confocal imaging, CLSM (Zeiss LSM800). SUN2-GFP expressing root (Arabidopsis) counterstained with FM4-64 (magenta). Overview (blend volume rendering and trimming, (c1). Close up on a row of nuclei (c2) or a single nucleus (c3) showing 3D reconstructions of SUN2-GFP labeling in a heatmap color mode. 3D rendering allows to visualise SUN2-rich regions forming a belt in 3D (c2, right panel, segmented nuclear surface in grey, SNU2-GFP max intensity signals in heatmap color) and discrete domains in the nuclear membrane (c3, arrows. xy section-top and partial projection-bottom). Image source KG. (d) Confocal imaging, Spinning disk (Visitron Systems GmbH Visiscope). H2B-RFP expressing root (Arabidopsis). Overview (max.projection, D1, grey, H2B-RFP. Background fluorescence pseudocolored in cyan) and detail of a single nucleus after image trimming at the same magnification (D2, max projection – top, orthogonal slices – bottom). Image source TD. (e) Multiphoton imaging (Leica SP8 MP). H1.1 RFP, H1.2-GFP expressing roots (Arabidopsis) mounted in 0.5xMS and imaged in water-immersion objective (25x, NA 0.9). Overview (blend volume rendering – left, orthogonal slicers middle and right panels). The imaging depth enables imaging nuclei throughout the root organ. Image source CB. (f) Multiphoton imaging & Hyvolution-based restoration (Leica SP8 MP). Same sample as in E. A single nucleus from the cortex region has been re-imaged at higher magnification (63x NA 1.3) and using the Hyvolution module (SVI-based deconvolution on the fly). This enables analysing H1.1 and H1.2 distribution as punctuate foci in euchromatin and chromocenters, showing H1.2 islands distinct from H1.1 regions. Image source CB.

In practice, optical microscopy allows for two signals to be resolved when they are separated by at least a distance approximately less than half the excitation wavelength. It corresponds to Abbe’s light diffraction limit approximated as d = λ/(2 NA) (reviewed in [49]). Thus, providing an optimised imaging design, wide-field and CLSM imaging of fluorescent nuclear probes can resolve structures in a ~ 200 nm range (for a fluorophore excited at 488nm and a NA = 1.4 objective). These typically correspond to ‘large’ molecular structures *e.g*. nuclear/chromatin domains, speckles or nuclear bodies (Figure 1). This resolution is achievable in the lateral resolution (x-, y-axis), even in fresh whole mount tissues, but particularly in superficial and/or optically transparent tissue layers (*e.g*. root and leaf epidermis, root tips). By contrast, the axial resolution (z-axis), although improved by CLSM, suffers from additional aberrations that can considerably reduce it to ~500 nm. Corrective lenses can recover resolution to ~250–300 nm.

Limitations in resolution obtained from conventional wide-field and confocal microscopes can be partially overcome by a method of image restoration known as image deconvolution. This method uses a mathematical-based treatment of signal information to remove the blurriness induced by light diffraction around each signal point (and described by a ‘Point Spread Function’, PSF). Image deconvolution can be applied post acquisition (*e.g*. using commercial or open source software), or ‘on the fly’, *i.e*. when image processing is integrated with acquisition (see below). In any case, the image signal needs to be oversampled by a factor of 2 to 4 in all dimensions according to the properties of the Fast Fourier Transform (FFT) [i.e. the Shannon-Nyquist sampling theorem] [38,49], highlighting the importance of sampling strategy consideration prior to acquisition. It is however possible to negate this requirement using a microscope that offers deconvolution at acquisition by decomposing pinhole projections into an array of detectors [50], *e.g*. Zeiss Airyscan).

While resolution of well-separated punctuate structures is less problematic, closely-related signals and/or poor image contrast obtained in thick and diffracting samples will often reveal the limits of the imaging setup. In this case, observed co-localisation should be interpreted with caution [39]. In addition, when assessing the respective localisation of two (or more) tagged proteins or dyes in the nucleus, it is important to control for chromatic aberrations using calibration beads of various diameters labelled with the relevant fluorescent dyes. Chromatic aberrations are inherent to the optics set (lens and illumination) and can produce up to 100 nm positional shifts between two channels in the lateral and axial dimensions [39,42,49]. The importance of careful microscope calibration prior to image acquisition was demonstrated by North who showed clearly the extent to which chromatic aberrations can lead to drastic misinterpretation of nuclear signal positions [42].

Recent advances in microscopy that enable resolution below the theoretical diffraction limit are becoming increasingly popular for imaging plant cells [46,47,51]. Depending on the instrument, super resolution microscopy (SRM) imaging can resolve nuclear signals ca. 20–90 nm apart. While not yet suitable for time-lapse imaging (due to a requirement for prolonged illumination and/or very limited imaging depth), some super resolution solutions are applicable for high resolution imaging of the nucleus in whole-mount plant tissue. In particular, versatile Structured Illumination Microscopy (SIM) [52] is a promising approach. Unlike other SRM approaches that require specific fluorophores, SIM is compatible with regular fluorescent probes due to its relatively fast acquisition rate [46,47,51]. SIM has been successfully applied to image the rapid movements of CRISPR-Cas9-tagged telomeres in the tobacco leaf epidermis [53]. However, the limited imaging depth of SRM imaging (*e.g*. 10–20 nm for 3D SIM) is the major obstacle to imaging nuclei in whole-mount plant tissues. Realistically, these approaches can only be implemented on isolated nuclei preparations to fully benefit from their resolving power.

### Imaging the plant nucleus in whole-mount fresh tissue

#### The benefits of imaging nuclei in whole-mount tissue?

The nuclei of plant cells are often described according to a canonical, cytogenetic model as a sphere encapsulating the chromatin organised along well-defined heterochromatin (chromocenters in specific species) and euchromatin compartments, a nucleolus, nuclear bodies and a nuclear envelope punctuated by nuclear pores. While such a model has the virtue of capturing relevant functional structures, it fails to convey the great diversity of shape, ploidy and structural organisation observed in different plant species, cells and tissue types and at different developmental and physiological stages. Although this diversity is well known and has been mentioned in several reports, it has not been systematically documented. For instance, in addition to plant cell nuclei that are spherical, they can also adopt rod- or lens- shapes, depending on cell type [54,55]. Their nuclear membrane shows complex invaginations [56], and at the chromatin level, the heterochromatin fraction as well as the cytological distribution of epigenetic modifications show high plasticity depending on cell type, physiological states and development and environmental cues [8,11,57–64]. Thus analysing cell-type-specific nuclear organisation is essential for understanding the diversity of functional organisation beyond a single canonical model. One approach is microscopy imaging of the nucleus within its tissue context: in whole- or semi-whole- mount organs.

Microscopic analysis of nuclei in living cells and tissues can be performed using conventional or advanced fluorescence microscopy instruments (Figure 1), with each approach presenting unique compromises on imaging depth and resolution. Wide-field (epifluorescence) microscopy, while suffering from a highly limited axial resolution due to out-of-focus light collection (Figure 1(a), see xz projection), remains well-suited for imaging the global distribution of nuclear labels, particularly in transparent tissues with limited thickness, such as in roots. Figure 1(a) shows an example of DAPI and H1.1-RFP staining in fresh Arabidopsis roots where whole-mount widefield imaging in 3D is still sufficient to resolve chromocenters at a level enabling the quantification of the heterochromatin fraction. Figure 1(b) shows an example of widefield imaging where axial resolution is significantly improved (compare Figure 1b2 with a2) via image restoration on the fly using the Optigrid system (see [65] for a review). Confocal microscopy implemented either in a point laser scanning or spinning disk system (Figure 1c,d) removes out-of-focus signal and thereby drastically enhances resolution in all dimensions. Figure 1c illustrates a good example of well-resolved nuclear membrane in whole-mount fresh roots expressing a GFP-tagged SUN2 protein; 3D volume rendering and plotting the signal on a heat-map intensity scale enables visualising a nuclear belt of higher intensity (Figure 1(c2)) and reveals discrete enrichment foci within the nuclear membrane (Figure 1(c3)). Confocal spinning disk imaging is an alternative to CLSM with fast acquisition suitable for time-lapse imaging and offering a reasonable axial resolution of subnuclear structures, particularly in optically accessible superficial tissue layers (Figure 1(d)).

Generally, and despite a few successful examples illustrated in Figure 1(a–d), imaging in fresh tissue faces the challenges of sample thickness and adverse optical properties. Cumulative light absorption and scattering throughout tissue layers increases optical aberrations and leads to drastic loss of spatial resolution and signal in deeper tissue. These issues defeat the goal of capturing nuclear features at the sub-micrometric scale throughout tissue layers, even when using objectives with high NA and lenses that correct for chromatic and axial aberrations. The advent of multiphoton imaging unlocked the potential for deep-tissue imaging (Figure 1(e), see the orthogonal sections throughout the root and close up panel), particularly when combined with image restoration. Figure 1(f) shows an example of 3D image restoration on the fly (using the commercial module Hyvolution, Leica microsystems, Germany) of root nuclei from the cortex layer. This approach resolves the intricate distribution of H1 variants in 3D within chromocenters and euchromatin (insets, upper right panel).

#### Considerations for sample-mounting

Plant tissues are best prepared fresh in a physiologically-friendly mounting medium, for example water or a growth-medium base (*e.g*. half-MS). The addition of a mild clearing agent such as glycine 1M can improve optical transparency without compromising fluorophore stability or localisation. It is recommended to avoid osmotic media and fixatives and to equilibrate the tissue in the mounting medium prior to imaging; some protocols even use slight vacuum infiltration of a mounting medium matching the refractive index of the tissue [66]. This delivers the additional benefit of reducing light-scattering air pockets in the tissue, but care should be taken not to stress the tissue, as this could lead to an accumulation of secondary compounds that may interfere with fluorescence imaging [35].

Testing different combinations of mounting media and incubation times is good practice, since these can each impact both fluorophore fluorescence and tissue penetration. It is also important to consider the pH, as this may affect the binding properties and/or localisation of the dye or tagged protein. This is particularly relevant for nuclear proteins with chromatin-binding properties involving weak interactions influenced by ionic strength. For example, GFP-tagged linker histones are destabilised at basic pH and consequently re-localised to chromatin-free nuclear regions (Baroux C, unpublished observations). Finally, temperature variations may also influence image quality. Although not formally investigated in (plant) cells, empirical observations suggest increased cytoplasmic streaming [67,68] and nuclear jiggling upon temperature variations, which can be explained by thermal convection motions in the cytosol. It is thus advised to equilibrate samples at ambient temperature prior imaging and, when possible, to use a controlled environment with a constant temperature (*e.g*. 18–20°C for plant tissues).

#### Capturing nuclei in shoot vs root tissues

As outlined earlier, plant tissues pose considerable challenges due to their optical properties. When appropriate to the biological question being addressed, roots are the tissue of choice for microscopic imaging of the nucleus, as they are relatively small in size and have minimal sample thickness. These features simplify both mounting and imaging processes. In addition, the low level of interfering pigments provides favourable transparency compared to other tissues. Together, these factors make it easier to capture the distribution of tagged nuclear proteins at high resolution using conventional microscopy, as illustrated in Figure 1. Note that in some cases, imaging roots at the horizontal position can be an obstacle, particularly if the study is focused on root growth or gravistimulation. To solve this issue, von Wangenhein and colleagues developed a confocal microscope setup for vertical sample mounting [69,70] .

Although more challenging, live imaging of plant nuclei in green tissues is feasible and strongly labelled structures can be well resolved, particularly in epidermal cells. Beautiful examples of dynamic movements of chromocenters have been captured in leaf epidermal cells using the LacO-LacI tagging system [71,72], and numerous examples of imaging nuclear speckles and chromatin in leaf tissues are reported. Tissue thickness and abundant pigments render imaging more difficult for low-intensity and/or cell-specific signals where long illumination times induce both photobleaching and stress-induced autofluorescence. In these cases, imaging nuclei in fixed, cleared tissues brings considerable benefits (see section 1.3, and 1.4).

Two-photon microscopy allows nuclei to be imaged in deeper tissue layers. Examples shown in Figure 1 demonstrate the high level of detail that can be obtained for nuclei from deeper layers, especially when combined with deconvolution-based image restoration. Though not yet routine, this approach deserves increased attention due to its ability to reveal tissue- and cell-specific nuclear organisation at a fine scale. Notable examples of studies benefitting from multiphoton microscopy imaging include measures of chromatin mobility in different root tissue layers [73,74], the capture of nuclear migration, karyogamy and histone dynamics during double fertilization in intact Arabidopsis ovules [75–77]. However, time-lapse imaging does not yet allow for subnuclear resolution in complex tissues, and capturing nuclear dynamics over time remains a major challenge.

### Imaging the plant nucleus in whole-mount fixed, cleared tissues

**Figure 2.**
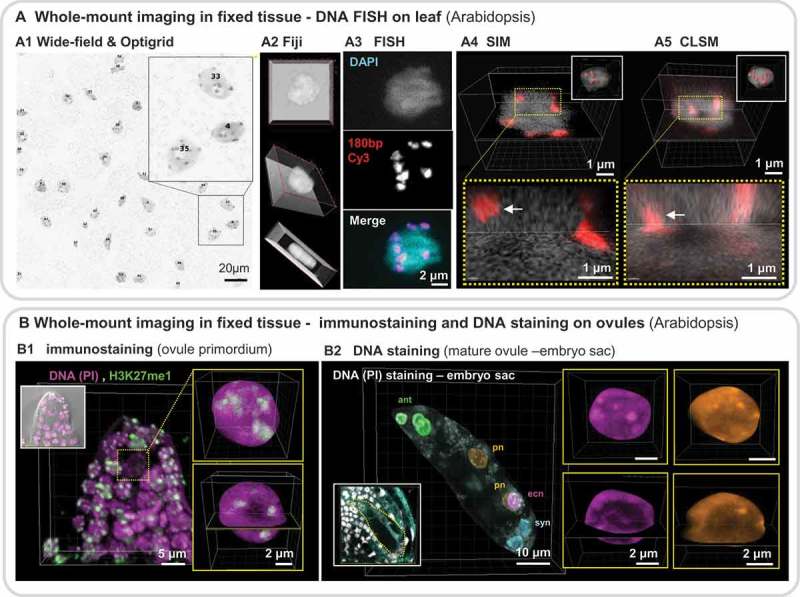
Imaging plant nuclei in whole-mount fixed tissue. Examples of imaging nuclei in 3D in whole-mount fixed tissues are shown for different nuclear fluorescent labeling: FISH, chromatin immunostaining, DNA staining. (a) Imaging nuclei in the leaf epidermis (Arabidopsis) at high resolution following DNA FISH. Overview of a leaf fragment, wide-field image stack of 6.5 µm^2^ (2048 x 2048 pixels) (a1) in transmission light. Nuclei are stained with DAPI, Close-up on subregions and individual nuclei. (a2) 3D projections using Fiji in a tripartite panel. (a3) Fluorescent *In Situ* Hybridization (FISH) decomposition into the individual DNA (DAPI) and FISH probe (180bp repeat oligo labeled with Cy3) and overlay (merge) – right panel. Comparison of image details obtained using structured illumination microscopy (SIM, Leica DM6000 & Optigrid, (a4) or confocal imaging (CLSM, Zeiss LSM800), (a5) of the same nucleus. The images show orthogonal slicers (top panel) and a close-up view (dotted yellow box, lower panel) showing the different resolution and image contrast, particularly at chromocenters (red). A 3D reconstruction with segmented nuclear surface (grey) and chromocenters (red) are shown as insets. Empowered by Imaris (Bitplane AG, CH). (b) Imaging nuclei in whole-mount ovules (Arabidopsis) at high resolution following immunostaining (b1) or DNA staining (b2). Ovule primordia (B1) or mature ovules (B2) were embedded in acrylamid, fixed, cleared, permeabilised, immunostained for a chromatin mark (H3K27me1, green, B1) and counterstained for DNA (PI, propidium iodide, magenta, B1) or stained for DNA only (PI, B2). Ovules were imaged by confocal microscopy (Leica SP2 and SP5, 63x Gly, NA 1.3) with 2–3-fold oversampling. The images were denoised but not deconvolved. b1 shows a max.projection (inset: overlay with the transmission DIC channel) and detail of the nucleus of the spore mother cell after 3D segmentation (yellow insets) as max.projection and with orthogonal slice views. This image quality allows for signal quantification and measurements of relative histone modification levels [91]. b2 shows a mature embryo sac before fusion of the polar nuclei (pn). The original image is shown in the inset (nuclei, grey. Reflection light, cyan). For this 3D representation, the embryo sac was manually segmented in 3D, as well as individual nuclei, to create 3D masks and corresponding channels identifying the polar nuclei (pn), egg cell nucleus (ecn), synergid nuclei (syn) and three antipodal nuclei (ant). Inerts on the right show max.projections and orthogonal sections of the ecn and one pn, showing high level of details in chromatin distribution.

Microscopy imaging of fixed *i.e*. non-living tissue is inherently limited to providing a snapshot of a nuclear process or state-of-organisation. Yet, it has the great advantage of enabling maximal 3D resolution (Figure 2). Indeed, fixed tissue can be subjected to optical tissue clearance in order to optimise light transmission and minimise autofluorescence. Clearance techniques date back over a hundred years [78] and the development of novel approaches that enable visualization of FPs and labelled antibodies within a tissue context continues to be an active area of research.

Strategies for tissue clearing include: physical dispersion of refractive fibres/polymers (for example using enzymatic digestion of the cell wall); molecular denaturation of light absorbing/dispersing compounds (for example denaturation of chlorophylls); and chemical methods based on dehydration and homogenization of the tissue’s refractive index through chemical infiltration of inter/intracellular spaces and organelles [79,80]. By reducing light scattering and absorption, these strategies render plant tissues more transparent, thus enabling optical sectioning methods for 3D imaging whilst avoiding destructive embedding and physical cutting steps. Published examples of plant clearing approaches include ClearSee and its derivative ePro-ClearSee [81,82], Transparent plant Organ MEthod for Imaging, TOMEI [83] and PEA CLARITY [84]. With the exception of ePro-ClearSee, all of these approaches effectively retain the fluorescence of transgenic proteins in whole mount samples. ePro-ClearSee enables antibody-mediated detection of chromatin marks by employing enzyme and 2-propanol treatments prior to ClearSee clearing to improve accessibility of antibodies and to minimise autofluorescence [85]. This method has been used successfully to detect methylated and acetylated histones, methylated DNA and the histone variant CENH3 in leaf samples from a diverse range of plant nuclei [81].

Alternatively, the successive use of methanol and xylene to infiltrate tissue samples has proven very effective for tissue clearing followed by Fluorescent *In Situ* Hybridization (FISH) and immunostaining. Whole-mount FISH was used to study chromosome arrangements using DNA FISH against repeats and chromosome painting in whole-mount young root tips and leaves [86,87]. Improved locus-specific labelling paves the way for 3D gene position analysis in whole-mount tissues [88]. Figure 2(a) shows an example of whole-mount centromeric repeat DNA FISH in leaf tissue. The benefits of an Optigrid imaging system are demonstrated with a near-isotropic image reporting on precise 3D centromere boundaries compared to CLSM imaging showing axial distortion (Figure 2a4 vs a5).

Whole-mount immunostaining also proves powerful for analysing and quantifying chromatin modifications and distribution patterns in specific cell types. Examples include chromatin studies in the gametes of ovules and anthers and in developing embryos in different species:- Arabidopsis, rice and maize [89–92]. Figure 2(b) shows an example of high-resolution chromatin imaging in whole-mount fixed ovules [89,91] showing immunostaining of H3K27me1, a heterochromatin-specific histone modification (Figure 2(b1)) showing punctuate foci colocalising with densely-stained DNA regions (Figure 2(b1) insets). Figure 2(b2) shows well-resolved chromatin structures of female gametes within the whole-mount ovule thanks to tissue clearing. In this case, 3D image segmentation (see section 2) improved information delivery by isolating each of the gametophytic nuclei into different (colour-based) channels and amenable to individual 3D inspection (Figure 2(b2), insets). Orthogonal slices show the high level of definition obtained for (hetero)chromatin distribution in the egg and polar nuclei (Figure 2(b2), insets).

Furthermore, whole-mount, fixed and cleared tissues can be embedded in a matrix to facilitate handling and multiplex sample preparation. Several protocols mentioned above describe an acrylamide mix based on an original development from the Bass lab [93]. Importantly, the appropriate acrylamide type and ratio should be chosen for ensuring optimal optical clarity for imaging [93]. Acrylamide embedded preparations are best imaged by confocal microscopy, but are not suitable for two-photon microscopy, where the high-intensity lasers appear to alter acrylamide pad-integrity (Baroux C, unpublished).

Nuclear labelling using antibodies or the application of chemical dyes on fixed tissue samples have a wide range of applications. They offer the benefit of circumventing the need for producing transgenic lines expressing fluorescent markers – a challenging and time-consuming task, especially for crops and non-model plant species. Another advantage is the versatility of these approaches; once established for a particular tissue/species, they can be readily applied to genetic mutants, natural accessions, landraces and cultivars. This enables investigations of genetic interactions and the effects of natural variation or domestication on cytogenetic traits.

Collectively, these methods that probe nuclear organisation in whole-mount samples are likely to remain invaluable for revealing knowledge of cell-specific nuclear composition and organisation in the future.

### Working with isolated nuclei – towards super resolution imaging

As discussed in the previous sections, imaging the plant nucleus in whole-mount organs or complex tissues has the great advantage that it can retrieve information at the cell-layer/cell-type level. Newly developed protocols and objective lenses make it possible to work with thick tissue samples (fresh or fixed preparations) and can deliver resolution down to ~250 nm. While this is sufficient for resolving ‘large’ nuclear domains, it clearly prohibits 3D fine-scale analyses of, for instance, co-localised protein complexes or genomic loci. For these questions SRM techniques are employed, but their limited imaging depth requires isolated and well-preserved nuclei. Another motivation for deploying approaches using isolated nuclei is the challenge of applying the fluorescence probe (dye, antibody or RNA/DNA hybridization probe) homogenously in a whole mount sample. Although helpful protocols exist [86–88,91] among others), scientists can be defeated by their complexity and lack of robustness. In this situation, it might be necessary to compromise on preserving tissue integrity by isolating nuclei or cells.

#### Nuclear isolation methods

**Figure 3.**
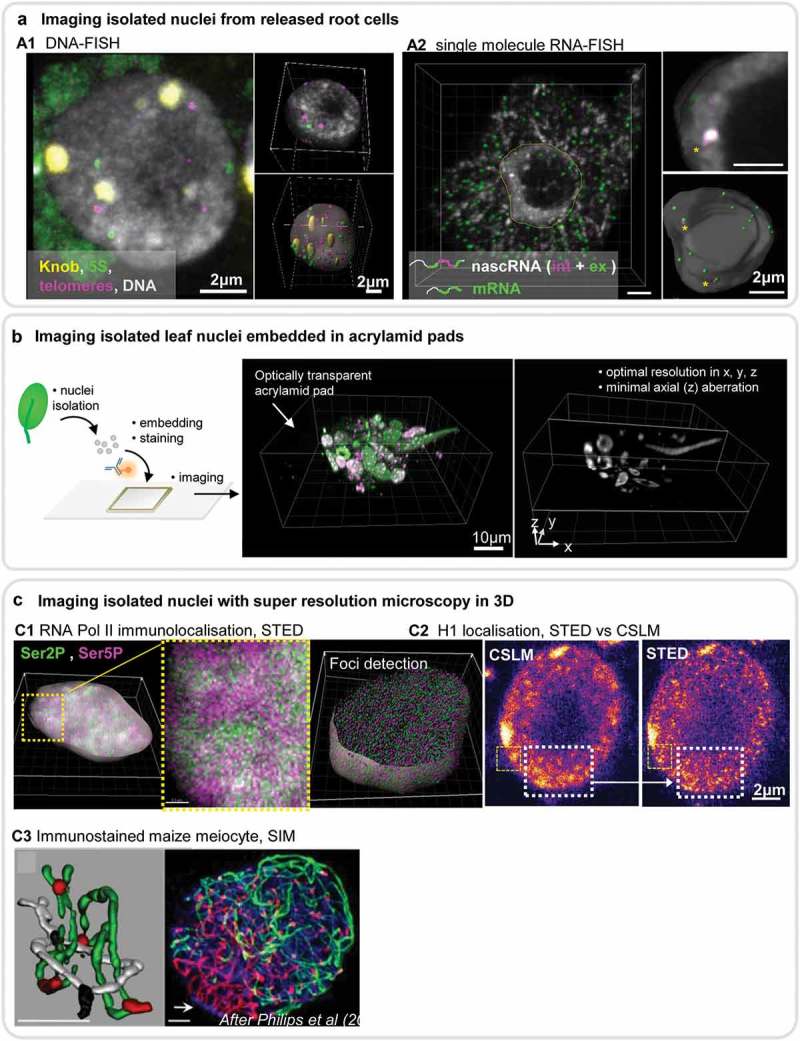
Imaging isolated nuclei at high resolution in 3D. (a) Imaging of nuclei from released cells: tissue squashes (*e.g*. root) produces nuclei with little deformation suitable for DNA and RNA FISH. (b1) DNA FISH on maize root nuclei showing a max.projection (main, left panel) of all channels as indicated in the legend (All probes are oligonucleotides: 5S rDNA-FITC, Knob-Cy5, telomere-Cy3; DAPI counterstaining). Chloroplast autofluorescence also appears in the green channel. 3D reconstructions and segmentation of the nucleus and signals are shown on the right. Image source INDEPTH training school (2018). (b2) RNA FISH on Arabidopsis root cells using oligoprobes PP2A-Quasar570 (magenta): PP2A intronic probe (2 spots); PP2A-Quasar670 (green): PP2A exonic probe (>100 spots), allowing to distinguish the nascent (nasc) from the mature messenger (m) RNA. DAPI counterstaining (grey). The stars (*) indicate two intronic signals co-localising with the exonic signals. Overview and 3D reconstruction and segmentations as explained in B1. Nuclei were imaged using a wide-field Leica DM6000 & Optigrid-based restoration. Image source INDEPTH training school (2018). (b) Principle of nuclei embedding for high quality 3D imaging. Plant tissues (*e.g*. leaf) are used to isolate bulk nuclei before embedding in an acrylamide (or other matrix) pad on slide and subsequent staining steps (see text for references). Images on the right shows the perfect transparency of the embedding matrix and intact nuclei enabling optimal, high quality imaging even with conventional microscopy imaging (here, CLSM). H1 and H2A/H2B labeling (green, magenta, image on the left) and DNA staining (grey, image on the right). Image source CB. (c) Imaging isolated nuclei with super resolution microscopy in 3D becomes feasible with the preparations illustrated above. Dark-grown seedling cotyledon nuclei were isolated and embedded for immunostaining against RNA Pol II isoforms (C1) or H1 (C2). c1 shows the distribution of RNA Pol II isoforms (Ser2P, Ser5P) in a segmented nucleus (SiR dye DNA counterstaining, grey « shell ») and close up displaying clusters of the distinct isoforms as reported before [97]. The segmented image shows spot detection (RNA Pol II foci) enabling future analyses of density and intensity distributions in 3D. Images acquired with Leica SP8 STED microscope. Image source RR & CB. c2 shows a comparison of signal resolution in a single plane of H1 distribution imaged by CLSM or STED. Note the higher signal contrast in the dashed-line region in the STED image. Image source CB. c3 reproduces a published panel of SIM imaging of maize meiocytes showing ZYP1(red) and ASY1(green) immunolocalisation with DNA counterstaining (DAPI, blue) and a 3D reconstruction of an interlock (left). Image source [108].

Commonly used methods involve extracting nuclei from whole tissues or preparing squashes (*e.g*. from leaf, inflorescence or roots) where a mixed population of nuclei can be prepared on slide. For instance, root squashes enable the recovery of intact nuclei suitable for 3D DNA and RNA FISH. Figure 3(a1) shows an example of multicolour FISH reporting on the 3D distribution of telomeres, 5S rDNA repeats and a maize-specific heterochromatin knob. Nuclei isolated from root squashes are also suitable for low-intensity single molecule RNA FISH (smFISH) (Figure 3(a2)). The development of this method has opened many novel possibilities: it allows one to investigate exonic *versus* intronic RNA [94] and nuclear-localised long non-coding (lnc) RNA [95,96].

The acrylamide pads described above can also be applied to isolated nuclei and provide excellent optical transparency, thus enabling the imaging of nuclei with near-isotropic resolution, even when using CLSM (Figure 3(b)). Flow-sorting of nuclear extracts offers nuclei selection. Sorting according to ploidy level is common practice and is mostly used to prepare a homogenous nuclei population to disentangle, for instance, tissue-specific chromatin organisation and composition from ploidy-effects (for instance [98]. In the case of the triploid endosperm seed tissue, ploidy-sorting enabled cell-type specific nuclei isolation and the description of cell-type specific chromatin and chromosome territory organisation [99,100]. Flow-sorting by the fluorescence of a nuclear FP tag constitutes another approach for cell-specific nuclei isolation. 

Cell-specific nuclear-tagging-based approaches such as the INTACT method (Isolation of Nuclei TAgged in specific Cell Types) are becoming increasingly popular due to the growing resource of tagged lines [101–103]. However, a systematic application for characterizing the diversity of nuclear architecture among plant tissues remains lacking. Once isolated, nuclei extracts can be fixed on-slide, but despite efforts to preserve 3D structure [104], they usually adopt a ‘flattened’ shape. To preserve intact 3D morphology, nuclei should be embedded, for instance in acrylamide pads.

#### Imaging isolated nuclei at high resolution

Imaging approaches enabling high-to-super resolution imaging, *i.e*. beyond the diffraction limit (~ less than 250 nm), include several SRM technologies such as STED, SIM and STORM (see also [46]).

**Figure 4.**
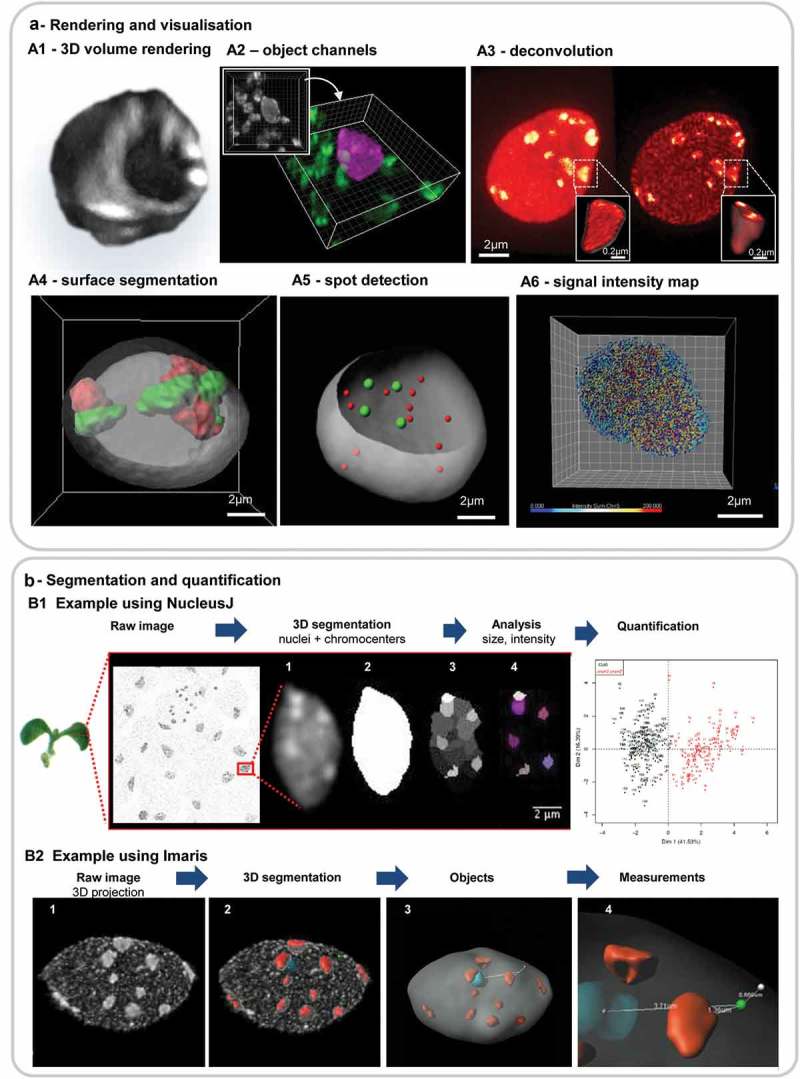
Examples of 3D image processing steps useful for qualitative and quantitative analyses of the plant nucleus. (a) Image rendering and visualisation. Reconstruction and segmentation-based visualisation of 3D image data for improved information delivery. (a1) Volume rendering (blend mode) with orthogonal slicers, (a2) object-based channel creation: following segmentation, the nucleus was separated from the surrounding material as distinct channel (mask) and pseucolored differently than the secondary channel. (a3) Image restoration by deconvolution (*Huygens, SVI*), DNA-stained nucleus imaged by STED imaging (a4) segmentation of the nuclear surface and chromosome territories using the surface function of *Imaris*, (a5) segmentation of the nuclear surface and FISH signals [centromeres (green), telomeric (red)] using the spot function of *Imaris*, (a6) segmentation of immunosignals (anti-RNA Pol II CTD-Ser2P isoform) using the spot function of *Imaris* and colored according to signal intensity (heatmap scale). Source of images: A2, A3, A6: isolated cotyledon nuclei embedded in acrylamide stained for DNA (DAPI, A2; SiR-Hoechst dye [107], A3) or immunostained (RNA Pol II-ser2P, A6); endosperm nuclei [A4, A5) DNA and FISH staining as described in [64, 108]. Reconstructions powered by the Imaris software (Bitplane). (b) Image segmentation and quantification. Two examples are shown that are used to segment and analyse chromocenter distribution in complex images reporting on bulk nuclei using the ImageJ *NucleusJ* plugin (b1), and to segment individual nuclei using Imaris *XTFISHInsideNucleus* plugin (b2). b1 Image acquisition of a cotyledon epidermal layer following DAPI staining using a wide-field microscope (Leica DM6000). The complete image contains up to 20–100 nuclei. (1) Each nucleus is individualized and (2) then subjected to segmentation. Segmentation of the nucleus is based on Otsu’s thresholding method. (3) Chromocenters segmentation is based on the watershed algorithm applied here to 3D images. (4) Finally, the user manually determines the threshold to be applied in order to obtain a segmentation reflecting the initial image: in this image six chromocenters have been manually validated by the biologist. Image source CT & SD. b2 Nucleus segmentation in *Imaris* can be done either semi-automatically (using the segmentation wizard for the different channels or automatically. 1. Max.projection of a DAPI stained nucleus (isolated, embedded as in Figure 3(a)) imaged by CLSM and deconvolved (Huygens, SVI) (2) segmented image: chromocenters (red), nuclear surface (grey), nucleolus (cyan). A theoretical FISH signal (green dot) has been added for illustration purposes. (3) Object detection and image rendering of nuclear bodies, (4) illustrates the possibility to compute the distance of chosen (or all) objects relative to each other and to the nuclear periphery (where a white, intersecting dot is placed). Image source courtesy of M. Ashenafi, image processing CB.

Although technically affordable, STED imaging is not yet widely used to image plant nuclei. Users may be hesitant to invest in new sets of antibody/fluorescent probes required for STED due to their resistance to the depletion laser while robustly emitting when excited at a distinct wavelength [105,106]. The increasing choice of fluorophores, relative easy use of the equipment, and convincing results obtained for intact (3D) nuclei embedded in acrylamide encourage the community to strongly reconsider this imaging approach: Figure 3(c1,c2) show examples of RNA Polymerase II (RNA Pol II) isoforms and H1 linker histone variant localisation. Figures 4(a3) and 5 show additional examples of DNA and immunostained plant nuclei. In these examples, STED imaging resolved nanoscale-sized DNA/chromatin and RNA Pol II clusters normally poorly or not distinguishable in confocal microscopy imaging.

**Figure 5.**
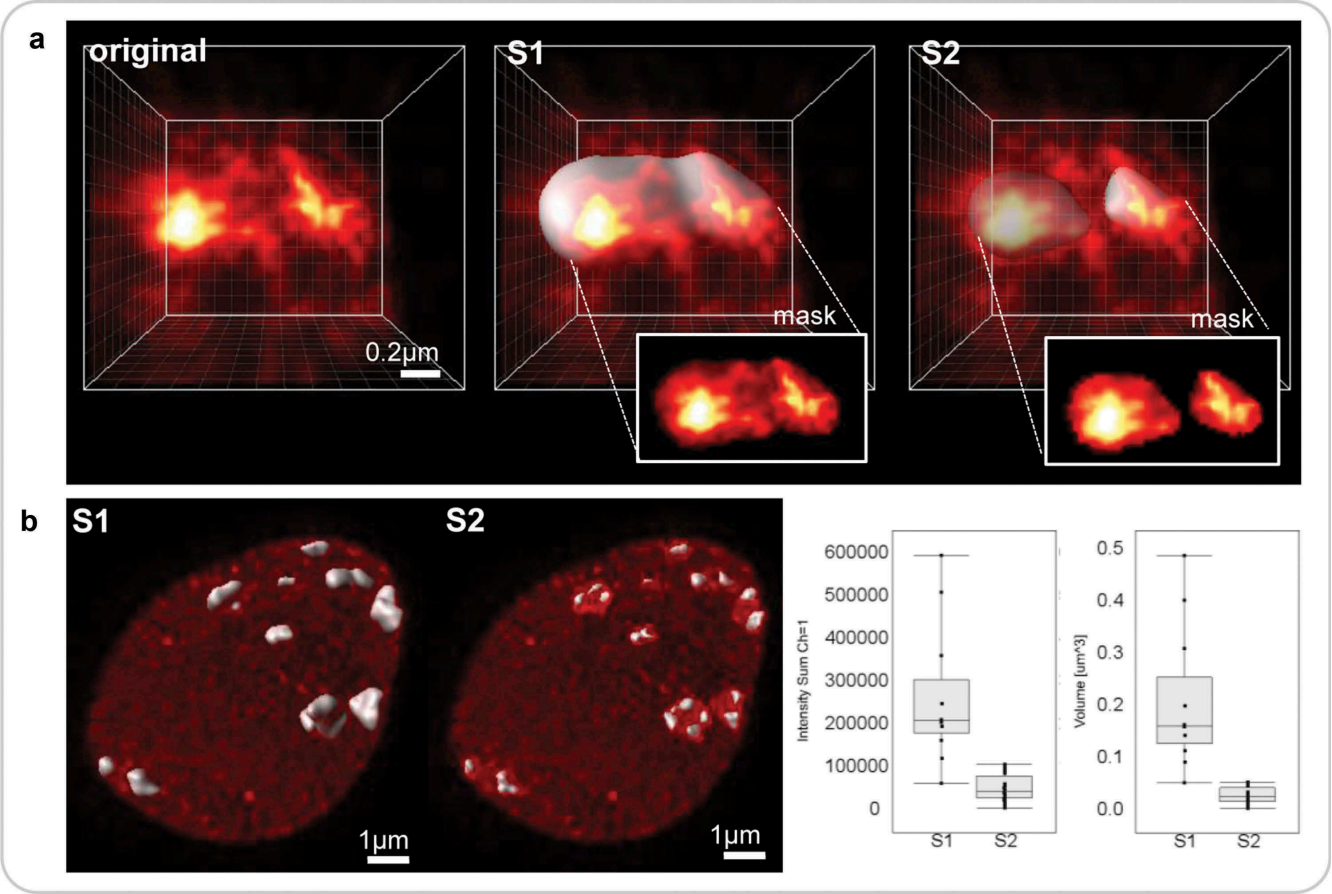
Impact of different segmentation parameters on downstream analyses of nuclear features. Segmentation parameters influence the shape and boundary of the 3D objects created and will impact downstream analyses in delivering different results of distinct biological significance. Segmentation parameters which may be question-dependent, should thus be defined at the beginning of each batch processing. (a) image detail of a chromocenter from a DNA-stained nucleus imaged by STED and restored by deconvolution (Figure 4A3): original, no segmentation; S1and S2, *Imaris*-based surface segmentation using the following parameters: background subtraction, largest sphere diameter = 0.4 µm, surface details = 0.1 µm, manual threshold value 24–97 (S1) or 59–166 (S2), without (S1) or with (S2) ‘split touching object’ function (seed point = 0.26 µm). S1 identifies a large, yet heterogeneously staining CC domain while S2 captures individual subdomains in the CC as shown in the insets (channel masks created on each surface). (b) The results from S1 and S2 are shown at the nuclear scale. Both methods yield different results with distinct biological significance (because they capture different object type) regarding the distribution of fluorescence intensity sum and volume of CCs (left and right graphs, respectively). *Graphs computed in ImarisVantage*.

Of all available SRM approaches, 3D SIM appears to be the preferred approach for imaging plant cell nuclei, due to its relative versatility and application with common dyes and FPs. For instance, 3D SIM, followed by 3D image processing, enabled visualisation of the synaptic progression in wheat meiocytes with unprecedented resolution [108] Figure 3(c3). 3D SIM recently contributed to understanding the roles of the topoisomerase TOPII and nucleoporin Nup1/136 in interlock resolution at meiosis in Arabidopsis [109].

STORM/PALM is being developed to image down to single molecules in isolated nuclei [110], but this is not yet routine practice. This is in part due to the requirements in sample preparation (sensitive redox scavenger buffers, ‘blinking’ *i.e*. photoactivatable fluorophores, preparation of nuclei on molecule-free coverslips etc.). Computational requirements downstream of imaging for the reconstruction of high fidelity signal distribution [46,110] present additional constraints.

3D SRM methods have yet to be applied routinely, but now is an opportune time for specific efforts to be made by the community to develop robust protocols. When used in conjunction with cell-specific nuclei isolation, they could offer enormous potential for future plant nuclear architecture studies.

### Perspectives

Nuclei isolation brings the power of large-scale, quantitative and high-to-super resolution analyses. Currently, SRM imaging of plant nuclear structures is in its infancy, but is eminently positioned to validate a functional model of the plant cell nucleus at the nanoscale level. Some key goals include:- imaging functional domains – LADs, NADs, knots and KEES, small-packaging units equivalent to animal TADs that are hypothesised by probabilistic models of interaction frequencies [5,15,18,111]; imaging the molecular connections between the chromatin, the nuclear periphery (matrix and envelope) and their supposed continuity with cytoplasmic domains [1]; and capturing fine-scale dynamics of nuclear architecture (*e.g*. spatial movements of gene loci, transcription complexes or nuclear bodies) in response to environmental signals or developmental cues [3,10,13,64]. For this, future efforts should focus on robust methods for the isolation of intact nuclei and computational solutions to exploit the complex data offered by SRM in 3D, including mathematical modelling to transcend purely qualitative analyses (see discussion in section 2).

As a complementary approach, nuclear imaging in whole-mount remains indispensable. While successful cases were cited in this section, whole-mount approaches remain challenging not only at the imaging stage but also at the level of probe and reagent application, where variable effectiveness across different tissues and plant species repeatedly requires protocol optimization and customization.

The major issue of imaging-depth can be largely overcome using multiphoton microscopy. This deploys higher wavelengths to provide better tissue penetration of excitation light, and new-generation detectors provide enhanced sensitivity. In combination with optical clearing approaches compatible with nuclear stains and antibodies, we are getting closer to imaging nuclear architecture in 3D, at high resolution and in whole-mount tissue.

Additionally, real-time or post-acquisition image processing is becoming more user-friendly and computationally affordable (for modest image sizes). This offers the considerable benefit of improving image resolution for traditional (not SRM) approaches. However, development of dynamic live imaging as a routine standard methodology will require extensive imaging technology innovation to meet the need for greater working depth and high spatial and temporal resolution. For transparent and/or superficial tissues, customized platforms are being developed as well as processing ‘on the fly’ to correct for sample drifts, as discussed in the next section.

## Image processing: aims, possibilities, and where efforts should focus

As reviewed in section 1, the achievable level of resolution in 3D microscopy greatly improved over recent years, thanks to new protocols optimized for plant cells and new microscopy imaging technologies. As a basic statement to introduce this section, it is important to understand that the best image processing results will be obtained with high quality images where efforts have been made to achieve best possible image quality in terms of SNR and resolution, which are intrinsically linked [38]. A well-designed image analysis workflow will be required to quantitatively assess nuclear organisation parameters such as nuclear morphology, chromatin domains stained with DNA dyes including eu- and heterochromatin compartments, chromocenters, nuclear bodies and FISH or immunolocalisation signals. In addition to discussing open source *versus* commercial solutions and manual *versus* batch processing, we also consider deep learning-based methods. It is anticipated that the recent technological breakthrough made in this area will supersede existing image processing methods in the near future.

### Aims of image processing for 3D analyses of plant nuclei

Image processing may be used in two ways: (i) to enhance certain image properties for better data visualisation, hence aiding information interpretation or (ii) to compute image features that can subsequently be analysed. Both applications are illustrated in Figure 4. The reader is referred also to additional literature for a general introduction and/or in-depth comparison of image processing methods. One example is Uchida’s review offering a good pointer to neophytes to choose the appropriate method depending on the aim, an overview of image enhancement and segmentation methods and flowcharts illustrating possible image processing combination etc [112]. Another good introduction is the monograph series of Burger and Burge [113]. We discuss below, with examples in Figure 4, some approaches that are currently under-exploited but highly valuable in the field of plant nuclear architecture studies.

***Image enhancement and rendering*** increases the possibilities to communicate relevant information from complex datasets. Image reconstruction and 3D rendering are useful for data presentation and interpretation. Image series can be represented with surface and volume features, on full or partial projections, using customized slice modes and rotations among the multiple possibilities offered by image processing software. Some rendering examples are illustrated in all figures and emphasized in Figure 4(a). Several open source and commercial software packages are available and user-friendly for the plant nucleus research community, as reviewed elsewhere [44]. For image enhancement, images can be processed to increase the contrast between low and high signal intensities, reduce technical noise and enhance feature (edge) contrasts to emphasize distinct features relevant for interpretation (example Figure 4(a3)). Some examples of algorithms implemented in classical image processing software are based on the concepts of contrast-limited adaptive histogram equalization (CLAHE) [114], colour deconvolution [115] or image enhancement by edge detection. Fluorescence microscopy images typically benefit from denoising approaches [116], particularly when the image is corrupted by shot noise (*i.e*. discrete electronic noise produced by photon detectors and amplifier devices) often referred to as ‘salt and pepper’ background. Several algorithms are available as ImageJ plugins [117] or as denoising/smoothing filters in commercial image processing software. These image processing techniques should however not be applied prior to signal quantification as they will modify intensity distributions.

***Image segmentation*** of nuclear signals detected as distinct objects is used to characterise them in terms of their geometrical features (size, shape, smoothness) and their number and positions, either relative to each other or in the image coordinate system. For instance, measuring the size, shape and distribution of chromocenters in Arabidopsis nuclei has been a useful indicator to elucidate the role of nuclear envelope proteins or histone chaperones in nuclear morphology and chromatin organisation [118–120] (Figure 4(b1)). Furthermore, once signals-of-interest are segmented, their relative distribution, *e.g*. in the nuclear space or relative to each other, can be computed (Figure 4(b2)). Such an approach was used to show that chromocenters are distributed in a non-random manner suggesting a spatial repulsion component [121]. Yet this approach is not routine for plant cytogeneticists. It deserves a broader use to empower quantitative analysis of plant nuclear organisation. In addition, there is an increasing interest in analysing the positioning of loci relative to chromosome territories or to the nuclear periphery to establish the functional link between gene activity and locus positioning. Yet, analyses have so far considered only 2D nuclei models [16,23,122]. To address the question in the real 3D space of the nucleus it is necessary to retrieve spatial distance information. Some image analysis solutions are beginning to be proposed for this [123], and recent 2D spatial positioning scoring systems [16] should be developed in 3D in the near future. Recording the relative position of transcription loci, topological domains, peripheral domain proteins etc. would allow for spatial statistics calculation and mathematical modelling. This in turns offers immense potential to unveil novel organisation principles in the plant cell nucleus.

### Common 3D processing options for plant nucleus studies

#### Image restoration

The imaging process unavoidably introduces a convolution of the signal emitted from the sample with the so-called Point Spread Function (PSF). The effect is a blurring of the signal that is inherent to the optics of the instrument. Deconvolution is a mathematical (image processing) operation that inverts the convolution from the imaging process to restore the original image. To perform deconvolution, the PSF is either calculated or measured experimentally using fluorescent beads imaged using the same parameters as used for the sample. Image deconvolution is a mature image enhancement technology available for the experimentalist (reviewed by [124]. Commercial solutions are available for 3D image deconvolution, such as *Huygens* (Scientific Volume Imaging) and *Autoquant* (Media Cybernetics), as are open source plugins like *Iterative Deconvolution 3D or DeconvolutionLab2* (ImageJ or Fiji) [124].

To obtain high image contrast and resolution after deconvolution it is important to acquire images with 2- to 3-fold oversampling (as described in section 1). Deconvolution has become one of the key image-processing tools not only for widefield microscopy but also for confocal-based imaging and SRM (Figures 1, 3, 4(a3). Image restoration by deconvolution is an important step before any quantitative measurement, as it corrects for voxel intensity distributions in the image, precisely defines the centre of mass of discrete signal foci (an operation at the basis of object segmentation), enhances signal contrast and in turn influences the resolution of signal distribution patterns.

#### Image segmentation

Segmentation is one of the key processes in bio-image analysis required to delimit the object-of-interest from the background: the nucleus, nucleolus, nuclear bodies or punctate signals such as those from FISH or protein immunostaining. Segmentation generates a mask consisting of a binary image delimiting the object-of-interest in the raw image. The challenge is to define an accurate segmentation methodology, or at least an approach that enables segmentation of biologically relevant features. Several segmentation methods (reviewed by [125] are available and the user should determine the method best-suited to the object-of-interest. We describe here the simplest and most frequently used segmentation methods and refer briefly to others.

Classical ***threshold-based methods*** consist in identifying a given pixel intensity level (defined as a grayscale value) that allows for separating the object-of-interest from the background. Historically, one of the first thresholding techniques, the Otsu-based method, considers the image as being composed of two classes of pixels corresponding to the background and the object. The algorithm thus attempts to separate the pixels into two distributions classes with minimal intra-class variance and maximal inter-class variance [126,127]. This method works well if the object itself shows minimal contrast variation (*i.e*. homogenous labelling) and if individual objects are well separated from one another. The method is therefore not suitable for touching objects. At present there are about 15 standard approaches for threshold-based segmentation [128], but most neglect the spatial information present in the image. Another limitation appears in an automated batch-processing mode. The variability in pixel (or voxel) intensity distribution and ranges across image datasets makes it difficult to apply the same threshold to all images; the user is then prompted to define a new threshold value for each image.

As threshold-based segmentation typically results in under-segmentation, where two closely related objects, for instance two chromocenters in a nucleus, are segmented as one, a necessary next step is to apply a ***Watershed*** algorithm to split the fused objects. The *watershed* is based on a topographical interpretation of the grayscale image as terrain of mountains and valleys; the algorithm interpolates boundaries between objects based on the continuity in intensity peaks. Small defects in the segmented objects are tolerable and they can be redressed either by applying a smoothing step before thresholding (*e.g*. Gaussian or median filter functions in ImageJ or Imaris) or by convolving the image with a mathematical morphology operation (*i.e*. opening or closing) [129].

There are many alternatives to thresholding-based image segmentation. An approach that can work for not very dense and occasionally touching objects is based on edge detection (*i.e*. ***boundary-based segmentation)***. There are many approaches for edge detection but most common are gradient-based, zero-crossings. As a second step of the approach, all closed regions are identified and, if necessary, some of them are merged. ***Region-based segmentation*** algorithms offer alternatives to consider in cases of complex signal distribution that are not easily segmented by the above-mentioned algorithms. *Region growing methods* are based on an initial ‘seed’ signal that grows as a connected shape on connected pixels of similar intensities. *Region splitting and merging methods* operate iteratively by splitting and grouping together regions with similar characteristics. Most common approaches to implement these segmentation methods are the active contours (snakes), the gradient vector flow and the Level Sets plugins available in ImageJ and Fiji. ***Clustering-Based Segmentation***, such as K-means or K-Ripley, is a fourth set of methods which divide the voxels into clusters such that voxels of one cluster are more similar to each other than to those of other clusters. This method has been applied for SRM images [130]. Finally, emerging ***Artificial Neural Network-Based Segmentation*** methods based on various machine learning approaches are very promising and are discussed in section 2.3.

Thresholding can also be used as the final step of a signal-enhancement approach. For example, an object segmentation method that aims to detect ‘bright signal foci’ in the image is spot detection. As segmentation results, spots can be used for downstream measurements or simply for representation, and can be very useful for object-tracking. Spot detection can be computed by different algorithms available in common image analysis software. They typically rely on finding the largest response of a convolution with a Laplacean or Difference of Gaussian operators followed by thresholding. Spot detection enables the segmentation of discretely-distributed signals and facilitates relatively simple further analysis such as their spatial distribution or their positioning relative to other objects.

#### Concrete examples for the analysis of the 3D plant nucleus

Figure 4 illustrates how image rendering, enhancement and segmentation can benefit the analysis of plant nuclear architecture. 3D volume rendering combined with orthogonal slicers allow the user to appreciate the distribution of hetero- and euchromatin in 3D (Figure 4(a1)); image segmentation separates objects from the same channel into two groups, either subjectively or objectively discriminated (Figure 4(a2)); image deconvolution resolves nanoscopic details, for instance in chromocenters and euchromatin such as in (Figure 4(a3)); the segmentation of FISH signals either as surface or spots allows one to clearly visualise their spatial distribution within the nucleus (Figures 3(a), and 4(a4,a5)); segmentation additionally classifies the objects (volume or spot) according to a given feature (nuclear size, shape or fluorescence intensity of the signal, Figure 4(a6)).

Classically, when studying heterochromatin organisation, intensity threshold-based segmentation, complemented with watershed-based object splitting, is being used satisfactorily to segment the nucleus and the chromocenters in 3D images of whole mount tissue or isolated nuclei. This can be done using either the NucleusJ plugin (Figure 4(b1)) [131] or Imaris (Figure 4(b2)), the latter being successfully used via a batch-processing mode [123]. Imaris additionally possesses a manual tool for surface splitting and merging that allows to correct for possible under- and over-segmentation of objects *e.g*. of chromocenters; this step can prove very important in downstream measurements of chromocenter volume, number etc.

The difficulty posed by the nucleolus in nuclear segmentation is worth mentioning here: in classical DNA staining, the nucleolus exhibits very low intensity values compared to the rest of the nucleoplasm and requires specific adjustments for segmentation [123]. Furthermore, because the nucleolus can be close to the nuclear periphery, artificial engulfment can be produced in the course of segmentation, leading to an underestimation of the nuclear volume and degradation of nuclear morphology parameters. In this case, manual segmentation – where the contours are drawn by hand – may be preferred over threshold-based segmentation.

As mentioned earlier, mathematical and spatial statistics approaches urgently need to be deployed to transcend simple qualitative analyses and unveil spatial organising principles in the plant cell nucleus, such as specific pattern distribution, clustering effects and other spatial relationships. While such approaches are being developed, they cannot yet be easily implemented by non-specialists and future efforts should hence be devoted to translating such spatial statistics analyses and reporting in a user-friendly interface. The structuring of bio-image analysts recently undertaken in Europe within the NEUBIAS network will undoubtedly make it possible to better meet this expectation (http://eubias.org/NEUBIAS/).

#### Note on segmentation validation and data evaluation

Several difficulties are posed by segmentation, particularly for first time users. Depending on image quality, signal contrast and threshold/quality parameters set by the user, segmentation will define object boundaries that need to be assessed by the biologist. This step involves subjective decisions and the user faces questions such as ‘does this object make sense or should it encompass more signal and/or the neighbouring object’? An example is shown in Figure 5 where two sets of segmentation parameters are used to segment large chromocenters (S1) or substructures that are closely located spatially (S2) (Figure 5(a)). This approach has two distinct outcomes influencing downstream quantifications, such as chromocenter number, volume and fluorescence intensity (Figure 5(b)). Hence it is important that the user defines clear criteria guiding the set of parameters. There are no good or bad criteria, as long as they are justified (for instance, large *vs* small heterochromatic domains, S1 vs S2 in Figure 5). Such subjective criteria are also employed when drawing contours manually, for instance when generating so-called ground-truth masks used for evaluating the performance of automated segmentation [132]. In addition, when doing batch processing, it is important to inspect a random but significant number of images to control for segmentation accuracy with respect to the user’s criteria.

The benefit of image segmentation is to create 3D objects that can be used for quantitative analyses of object number, size, shape and signal intensity (among other criteria) and for spatial measurements, *e.g*. distance between objects/relative to the nuclear periphery. Importantly, data analysis should then follow conventional principles (‘Statistics for Biologists’, www.nature.com/collections/qghhqm). This includes data normalisation, verification of the data distribution and evaluation of the variance. Examples of normalisation include expressing the fluorescence intensity of a given antibody relative to that of another, co-localising antibody or dye [91], and the distance between objects relative to the nuclear size/diameter. The type of data (discrete vs continuous) and the shape of the distribution (*e.g*. Gaussian or non-Gaussian) will also guide on the appropriate statistical test.

### Examples of customized image processing workflows

Choosing the best segmentation method is not an easy task and depends on the image. We strongly recommend discussing with specialists in digital image processing before making a definitive choice and to test several segmentation methods. We provide below a few case studies to illustrate the complexity for biologists to solve their experimental questions.

#### Manual image processing – customized solutions to specific problems

##### Manual segmentation for cell-specific chromatin analyses in whole-mount tissues

We present here briefly an example where manual image segmentation was chosen for generating robust and trustworthy quantifications. The aim of the study was to measure the levels of various chromatin modifications in the female meiocyte precursor (Spore Mother Cell, SMC) compared to neighbouring somatic cells. Whole-mount immunostaining was performed with DNA counterstaining on semi-whole-mount, embedded Arabidopsis ovule primordia [90,91]. Because the SMC shows very distinct nuclear size, shape and chromatin density distribution, the application of an automatic, threshold-based segmentation method failed to efficiently capture the SMC compared to surrounding nuclei. Instead, the SMC nucleus as well as 6–8 neighbouring nuclei were manually segmented using manual contouring in the Surface function of Imaris. This Imaris function benefits from a robust interpolation algorithm enabling the user to draw only 6–8 contours per nucleus instead of every single plane, which would be ~30 contours. The object parameters of interest (here nucleus size, sphericity, intensity sum in all channels) were exported in .csv format for downstream analyses. The operation was repeated for several independent images in wild-type and mutant and over different developmental stages. For the analysis, double normalisation was used: chromatin modification levels were expressed as a ratio of antibody:DNA signal intensity sums and this relative level in the SMC was itself expressed relative to that of the neighbouring nuclei. This had the benefit of buffering variations between images and experiments. This protocol is available as a video tutorial [133].

### Semi-automated batch image processing

In the past years, workflows based on open-source or commercial software have been developed and made available to the plant nucleus community to facilitate large scale measurements of nucleus shape and size, to quantify chromatin organization or to measure the position of a given fluorescent signal within the nuclear space. We describe here two alternatives, one developed for the open source platform ImageJ, and another for Imaris, a commercial solution for 3D visualization and image processing (Bitplane, CH).

The first example is NucleusJ (Figure 4) [127,131] an ImageJ plugin coded in Java language. It includes all the necessary steps to process images of nuclei, to perform various analyses and to provide several quantitative parameters to describe the original image (Figure 4(b1)). The ImageJ platform was chosen because it is open source and among the most popular tools in Life Sciences for storing, processing and analysing images [134]. Starting from 3D image stacks, NucleusJ automatically delimits the nuclear boundary by a modified Otsu segmentation method [126] based on the definition of a threshold. Chromatin domains such as chromocenters are segmented by partitioning the nucleus using a watershed segmentation [135], here applied in 3D, and by manual thresholding a contrast measure over the resulting regions. NucleusJ then provides a set of parameters including shapes and sizes of nuclei and size and number of chromocenters as well as their positions in the nucleus relative to the nuclear periphery. Using NucleusJ, we successfully analysed 3D nuclei from various plant tissues (root and cotyledon) of wild type and several mutants [118,119,136]. We also found that alteration of nuclear morphology quantified by NucleusJ is associated with transcription of heterochromatic sequences that are usually silenced [119].

The second example is the XTFISHInsideNucleus [123], a plugin developed in Python and implemented in the proprietary software Imaris. This plugin performs segmentation of the nucleus, nucleolus and punctate signals (such as FISH signals) in the nuclear space in a batch-compatible mode (Figure 4(b2)). The segmentation procedure is based on thresholding and applies gaussian filters to smoothen the image. The user is prompted to adjust these parameters during the workflow, thus enabling an adaptive process. Distances are then computed between the punctate signals converted into spot objects and the nuclear periphery, the chromocenters and the nucleolus. Such a processing workflow now enables determining the spatial distribution of discrete foci (*e.g*. genomic/FISH signals, transcription factor detected by immunostaining) or objects (*e.g*. chromocenters, nuclear bodies, chromosome territories etc.). This approach is timely and relevant for studies addressing the role of spatial positioning in gene regulation [16,23].

Both approaches described here define objects within the nucleus but apply different segmentation methodologies. NucleusJ segmentation relies on the connected-component principle to define an object as a set of voxels connected to each other. As voxel size is known from the image acquisition system, NucleusJ computes the theoretical object volume (µm^3^). After segmentation of the nucleus, XTFISHInsideNucleus calculates an average intensity of voxels contained within the nucleus and determines sets of voxels over (FISH spots) or below (nucleolus) this threshold. Object size thus depends on a ratio between average intensities. These two different approaches (volume versus intensity) probably do not yield similar object size. Benchmarking of the two plugins using fluorescent beads of well-known size and a common plant nuclei dataset will determine the accuracy of object size definition by the two methods and its potential impact on the computed distances/volumes.

These customized image analysis software packages and plugins pave the way for analysing thoroughly the spatial positioning of nuclear domains, as demonstrated for chromocenters [119,121], but also for the spatial positioning of loci and transcribed regions (*e.g*. in combination with 3D DNA and RNA FISH [88,94,96,123], or of other nuclear factors of interest. In other words, these approaches offer new ways of analysing the 3D organisation of plant nuclei that deserve a more systemic application in the community to gain insight into the yet poorly described spatial and functional principles.

### Breakthrough solutions for image processing of 3D nuclei

#### Avenues of opportunities with deep learning

Initiated more than 60 years ago, the use of Artificial Intelligence (AI) in the image analysis community is gaining great momentum. AI refers to the simulation of certain forms of (human) intelligence by computer systems following a series of processes including learning, sorting and self-correction. In 1990, Machine Learning (ML), a subdomain of AI, arose as a process giving computers the ability to learn without being explicitly programmed for it. For image processing, ML algorithms must be trained using annotated images or by providing ground-truth images; because of learning, the quality of ML-based image analysis improves with the number of presented examples. There are more than 15 learning methods in ML including random forest, Bayesian networks, support vector machines (SVM) and deep learning (DL) among others. The emergence of Graphics Processor Units (GPU) drastically accelerated the development of DL applications by reducing processing times of complex operations from several weeks to just a few days. DL is useful because it saves the programmer from having to perform the function specification tasks (defining the characteristics to be analysed from the image) and the optimization (how to weigh the data to provide an accurate prediction) as the algorithm does both. Through this process, it is now possible to operate automatic segmentation of image elements or image restoration with increasing computational efficiency. DL algorithms are recent developments opening unprecedented avenues in bio-imaging [137,138]. DL-algorithms specific to nuclear segmentation in 3D images are also beginning to emerge, as offered for instance in Cell Profiler 3.0 [139]. Further development will be required to produce tools specific to restoring or extracting subnuclear features for the purpose of building 3D nuclear organisation models.

#### Image restoration

As discussed in section 2.1, image restoration is an important step prior to image processing (segmentation, classification, quantification etc). Current algorithms reach their limit as soon as the acquired image drops in quality, with very low contrast and SNR dramatically blurring structures of interest. This is the case in challenging time-lapse imaging where temporal resolution and organism viability necessitate a low photon budget that compromises spatial resolution. A recently developed DL algorithm working on a ‘content-aware’ -based concept remarkably restores images acquired with up to 60-fold fewer photons than the considered minimum for downstream processing, reaching near isotropic resolution in images down-sampled up to ten-fold [140]. Similarly, a customized and well-trained DL was recently reported to restore images acquired with a conventional microscope with a resolution approaching that of SRM [141]. Of interest for this review, the demonstration includes the restoration of immunolabelled histone H3 patterns imaged under CLSM to a STED level of resolution [141]. The possibility to probe the 3D plant nucleus with conventional microscopy and yet achieve SRM levels of information opens exciting opportunities that have yet to be exploited by the community.

#### Intelligent segmentation

Whatever the segmentation methods applied, having an accurate and generalizable solution for segmentation is urgently required for automated processing of large datasets, which are key to evidence-based discoveries.

ML/DL algorithms could be the key to achieving an ‘intelligent’ segmentation where best parameters can be automatically adapted on an image-basis. Trainable Weka Segmentation (TWS) and Active Segmentation [142], now implemented in Fiji, have recently presented new opportunities for analysing complex datasets [143]. The tool relies on learning the properties of the objects to be segmented via examples presented to it. The software is then trained, and the user allowed to improve the classification by adding new characteristics or examples. For instance, in Active Segmentation, the image regions presenting the objects of interest are convolved with a series of multiscale filters, which are then used to train the model to classify the objects. Cell segmentation can be implemented with the help of nuclear segmentation [144]. ILASTIK [145] and Cell classifier [139] are user-friendly tools combining ML and DL for interactive 2D and 3D image classification, segmentation and analysis based on fluorescent labelling (allowing the user to separate background, cell membrane, nucleus…). A future solution could involve using pre-trained networks coming from computer vision applications and applying additional training with the microscopic images of interest. This could be developed as a community effort of experts, which could either nurture a large pre-classified database of representative objects (*i.e*. nuclei in different conditions) or provide a sharing mechanism for pre-trained models.

#### 4D image processing

Ultimately, the expected and much anticipated progress in time-lapse imaging of nuclei in plant tissues and organs will generate novel needs in terms of image processing (see section 3). As discussed below, one challenge is adaptive imaging, involving nuclei tracking and stage correction on the fly, ensuring that the nuclei of interest remain in the field of view and in focus for the whole duration of imaging. Ideally, microscope providers should implement such algorithms or at least the possibility to implement image feature-based hardware control for such purposes. This would imply having the possibility to operate image processing during acquisition, an operation not currently implemented in many microscope control environments.

The second challenge concerns image segmentation, object detection and tracking over time, with ideally the possibility to visualise velocity and directional (movement) or growth/shape changes. Commercially available and open source image processing software offer partial solutions to these needs but the basic requirement to be met is the possibility to (i) register the images along the time sequence (*i.e*. align possibly drifting or rotating objects over the imaging process), (ii) unambiguously and automatically segment each of the time points. Since the computational requirements are becoming more affordable (either through high-end local stations or server-based processing environments) and hence not limiting, the problem is refocused on image quality (contrast and resolution) throughout time-lapse imaging. As explained in the first section, this is an experimental difficulty for which we are starting to see solutions, thanks to imaging platforms with increased speed and illumination efficiency for less phototoxicity. These are however not yet widely applicable and/or do not yet offer the greatest resolution for capturing fine-scale nuclear structures. Most likely, solutions will come from deep-learning based image restoration that provide a remarkable potential in reconstructing isotropic, high resolution images from images acquired with a low photon budget and undersampling conditions [140,141].

## In vivo imaging of nuclear dynamics: case-study and challenges

Working on fresh tissues or organs has the advantage of enabling dynamic processes to be recorded over time (also referred to as 4D imaging), bringing a unique insight into nuclear function *in vivo*. Time-lapse imaging, however, requires rapid imaging technologies to minimize photo-bleaching and preserve sample viability, while simultaneously acquiring sufficient data for downstream image processing and analysis. Prioritizing temporal resolution and sample viability is usually at the detriment of spatial resolution due to a necessary low photon budget and undersampling, which result in low SNR and hence poor resolution. Additionally, specific solutions are needed for adaptive imaging that corrects for sample displacement during a developmental process.

Thus the imaging design drastically changes whether nuclei are imaged as 3D snapshots (Static Live Imaging (SLIm) or followed over time in a Dynamic Live Imaging (DLIm) process. The sections below outline some key considerations and challenges and provide an outlook for fluorescence-based microscopy imaging of the nucleus in whole-mount plant tissue preparations from a recent case study.

### Dynamic live imaging

DLIm presents the greatest challenges for effective live imaging, as it has to fulfil several requirements throughout the imaging session: i) it must allow accessibility of the tissue/organ of interest; ii) there is a requirement to maintain the plant individual or sample alive by minimizing photodamage; iii) the objects-of-interest (here the nucleus) must be kept within the field of view and in focus whilst minimizing photobleaching. The difficulties associated with meeting these requirements differ depending on the imaging time required to capture the relevant nuclear process: 15–60 min for FRAP to 2–6 h for covering cell divisions and other short-term processes and up to several days for monitoring developmental dynamics – an experimental setup for the latter is yet to be reported for investigation of plant nuclear dynamics. Examples of time-lapse imaging of differentiating nuclei in growing roots are shown in Supplemental Files 1–4.

Accessibility of the cells differs depending on the organ-of-interest. Imaging nuclei in small, young seedlings (roots or cotyledons, leaf fragments) that can fit on a microscopic-slide coated with vital medium offers an accessible set-up for experienced microscopists. For larger organs, semi-*in vitro* solutions have been established: embryos, shoot meristems, floral buds, anthers and ovules can be detached from the plant and cultured in microscopy-optimized chambers [146–148,149] .

The imaging setup also affects sample viability, where excessive illumination and heat by-product can induce physiological stress-related autofluorescence and cellular damage. This aspect of DLIm requires extensive empirical optimisation and technical reviews are available that can help rationalizing systematic improvement [150]. Light sheet imaging can minimize phototoxicity considerably [151], but this approach has yet to be applied for the purpose of nuclear architecture studies. This is possibly because the numerical aperture of the objectives implemented on commercially available systems are typically unsuitable for resolving nuclear structures.

Concerning sample preparation, the challenges and solutions differ depending on the duration of imaging. Maintaining the sample alive for short DLIm, such as FRAP experiments (up to 0.5–2 h), is usually unproblematic and requires a simple physiological medium (*e.g*. half-MS). Imaging in a chamber with constant temperature of around 20°C for plant tissues minimizes thermal convection that can result in sample shift [152]. Another benefit of a constant, cool temperature is reduced nuclei jiggling inside living cells. For moderate (2–6 h) imaging, regular mounting procedures may be sufficient to capture biologically relevant nuclear dynamics [71,72]. For long-term imaging (>6 h), the setup has to implement light-, temperature- and mounting medium-control (preventing evaporation and oxidation over time) compatible with the physiological or developmental process being studied. Several suitable customized setups and controlled growth/imaging chambers have been described [69,70,151,153–155,156] . So far, reports on long-term DLIm in plants are cell- rather than nuclei-oriented, and imaging nuclei at high resolution will require adaptation of these methods. Recently, a combination of micro-dissection and two-photon microscopy has been used to acquire astonishing time-lapse images of male meiocytes in living anthers still attached to the plant [157]. This kind of method could appear helpful for DLIm of other organs that are relatively inaccessible, such as female reproductive structures.

Long-term nuclei imaging brings the additional challenge of tracking live nuclei that can move outside the field of view and out of focus during the experiment. Advances in the imaging of growing tissues, such as plant roots, have resulted in the development of different image processing-tracking tools that can correct the positioning of the microscope stage on the fly to maintain the sample in the field-of-view over several hours or days [69,70,158]. Recent tools customized to follow nuclear architecture dynamics in Arabidopsis roots are discussed below.

### Tracking moving nuclei during imaging

Tracking of moving objects is particularly challenging in microscopy; it requires following a moving object within a referential that is displaced over imaging time. While the first problem is addressed by object registration, the second problem is partially circumvented by frequent repositioning of the microscope stage over time and registration of time frames. This complex challenge is typically faced when imaging nuclei within a growing root: (i) the root tip, offering a reference point, is pushed away from the imaging field-of-view, (ii) the nucleus of interest itself changes position with regards to the root tip and (iii) with regards to neighbouring nuclei due to cellular elongation during cell differentiation.

Tracking methods for root tips have been published [69,70,159,160] . They function on the same broad principle of comparing successive time-points to predict the next position and re-adjusting a motorized microscope stage accordingly. However, a tracking solution that was both compatible with a spinning disc confocal system and suitable for sub-nuclear 4D imaging in our dynamic environment was missing at the time of our study. As the microscope’s proprietary control software was nonextensible, a new tracking program called TARDIS was written and is described in Supplemental data 1. In brief, the position of the stage was corrected whilst imaging with a 10 min interval by communicating vectors of readjustment calculated based on segmentation on the fly. Examples of time-lapse images acquired with TARDIS are shown in Supplemental Files 1 and 2 (7 h, 10 min time step). This experimental setup enabled us for the first time to capture dynamic processes including nuclear elongation, chromocenter movement and fusion in interphase root nuclei during cell differentiation. Despite its apparent complexity, we believe that this approach has unique potential to reveal live nuclear processes that cannot be captured by static imaging.

As a case study of environmentally-induced nuclear dynamics, remarkable changes in nuclear and heterochromatin morphology were observed in response to heat stress. Drastic chromocenter decondensation and depression of heterochromatin regions had been described previously in nuclei from roots subjected to prolonged heat stress [161], but the process was never observed live and on individual nuclei. Here, nuclei were imaged successfully over 30 h during which nuclear features, including size and heterochromatin content, was measured. A rounding of nuclei and a strong reduction of nuclear movement were observed within the cells upon heat stress treatment (Supplemental File 3), while nuclei under regular temperature remained elongated and exhibited high intracellular mobility throughout the observation (Supplemental File 4). Measuring RHF dynamics for individual nuclei confirmed a pronounced loss of heterochromatin condensation during heat stress (Bassler, Dumur, Mittelsten Scheid, unpublished). However, RHF values decreased over time in the mock condition and the extent of chromatin decondensation in the heat stress condition was lower than in control conditions. In this case study, difficulties were faced at two additional levels: (i) nuclei exhibited high mobility within the cells and frequently drifted outside of the field of view and focal range. This was solved by imaging with multiple fields-of-view followed by stitching prior to processing; (ii) decreasing fluorescence levels due to photobleaching over time which reduced the accuracy of RHF quantification. This suggested a remaining impact of the imaging conditions despite optimization of the imaging chamber, medium and sample illumination.

### A case study for supervised nuclear segmentation in a 4D time series

To investigate changes in nuclear architecture in response to heat stress, a fluorescent chromatin reporter was imaged in living roots with the challenging objective to follow individual cell nuclei in whole-mount tissue (Basler, Dumur et al, in preparation). We provide here a case-study whose goal was to monitor nuclear shape, size and heterochromatin content (chromocenters) in growing roots over the duration of the treatment (4D-imaging).

**Figure 6.**
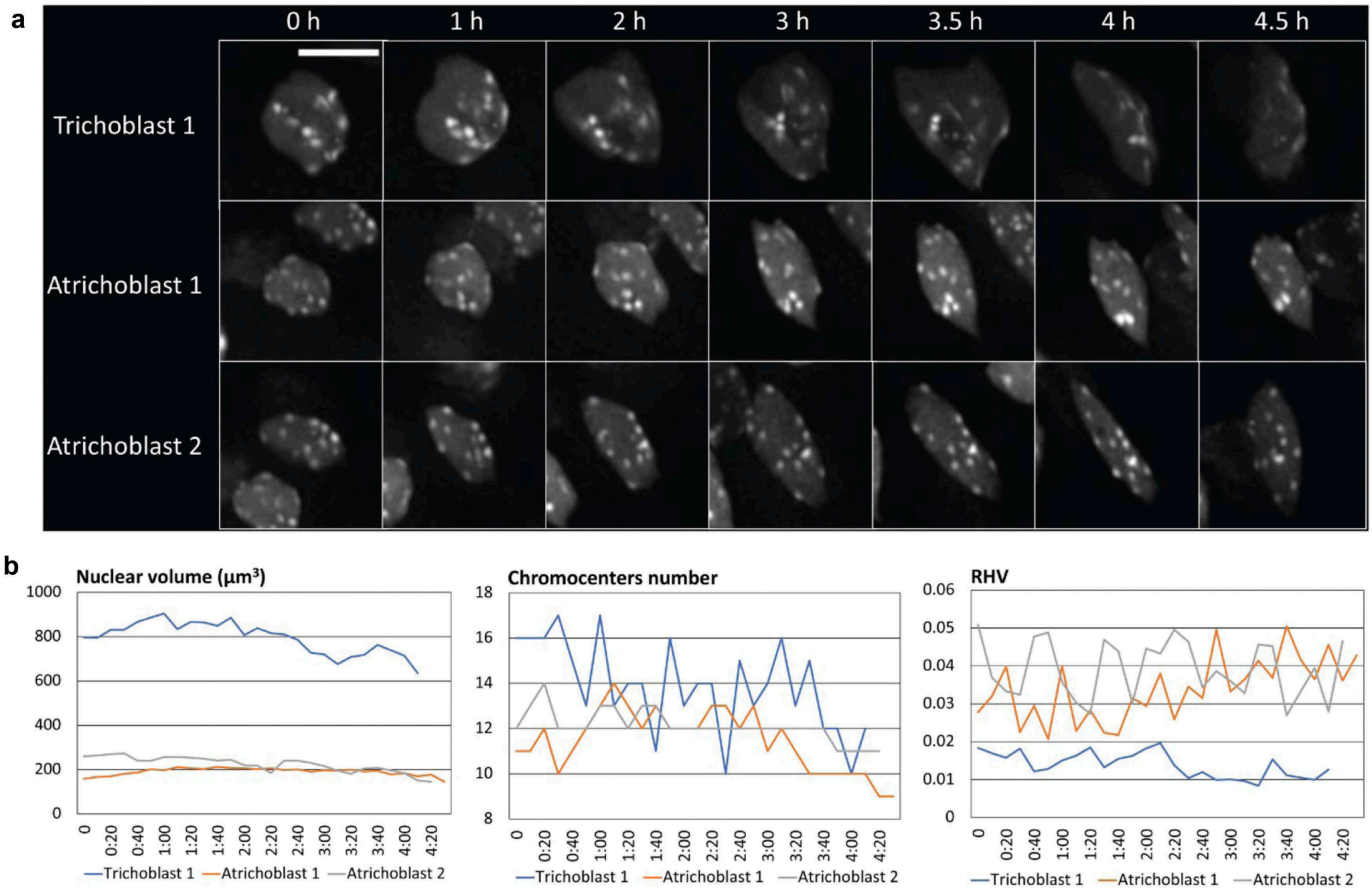
Live-tracking nuclei using the TARDIS pipeline. Growing, intact roots were mounted in physiological medium and imaged with a spinning disk microscope for 5 h. The field of view captured several nuclei, as shown in Figure 1(d). The TARDIS software allows for microscope stage repositioning and live nuclei tracking, facilitating downstream image processing aiming at capturing quantitative changes in nuclear organization. (a) Representative maximum intensity projection of three individual nuclei at indicated time points, scale bar = 10 µm. The last timepoint of trichoblast 1 correspond to 4 h 20 min. (b) Quantitative analysis of nuclear architecture: nuclear volume (left), chromocenter number (middle), and relative heterochromatin volume (RHV) measured from nuclei shown in (a) during the time-lapse experiment.

Several problems were posed by the experimental setup. Firstly, growing roots implied a displacement of the nuclei in the imaging field – an effective tracking method was thus developed for this specific experiment. Secondly, imaging of fresh tissue over several hours necessitated compromising spatial resolution to accommodate repetitive imaging and preserve both tissue integrity and fluorescence levels. As a consequence, the image contrast did not allow existing software to robustly segment closely-positioned nuclei as distinct objects. Thirdly, the diversity of nuclear architecture in growing roots and variability in signal intensities defeated batch image segmentation pipelines where non-adaptive thresholding impaired robust nucleus and chromocenter segmentation. To circumvent this, manual segmentation was opted for, together with manual processing of each dataset and time point. The nucleus and its chromocenters were manually segmented using the surface function of Imaris (Bitplane AG) with local thresholding and background subtraction. In 4D data-sets, each time point was analysed individually to better fit the segmentation to the images. The result for each individual segmentation was manually saved and parameters of interest of the segmented objects (size, density, intensity sum and mean etc.) were automatically script-sorted in an Excel sheet for further quantitative analyses. It allows for the monitoring of nuclear features over time, such as volume, shape, chromocenter number and potentially any other labelled compartment. In the example given in Figure 6, nuclei from two distinct cell lineages in the root epidermis were imaged over 4.5 h, corresponding to the root hair lineage (trichoblast) and non-hair lineage (atrichoblast). 4D stacks were processed to retrieve nuclear volume, number of chromocenters and relative heterochromatin content per volume (RHV). In this example we detected differences in nuclear volume between the two cell types and high fluctuations in the number of dense heterochromatin objects (collectively referred to as chromocenters). Yet the global RHV fraction did not dramatically vary over time. This suggests that the temporal variations in chromocenter number detected may correspond to rapid fragmentation/association dynamics of dense chromatin patches forming a mere constant (over this time frame) heterochromatin pool. This example illustrates the potential of time-lapse imaging and 4D image processing to reveal dynamic nuclear processes overlooked in static imaging.

This last example illustrates the potential of 4D imaging to meet the eminent objective in the field of plant nuclear biology to better understand how nuclear architecture changes in response to environmental stresses and in relation to changes in gene expression underlying physiological adaptation [13].

## Concluding remarks

By harnessing over a century’s worth of investigations, from hand-drawn documentation of traditional cytology to computer-based modelling of subnuclear dynamic processes, we have gained considerable understanding of the functional organisation of eukaryotic cell nuclei, particularly their 3D architecture and composition. But since progress in plant nuclei studies have lagged behind animal cell research, many open questions remain that are unique to this system. A pertinent example is addressing the role of nuclear and chromatin organisation during cellular reprogramming in response to environmental cues and in plant cell totipotency. Concerted efforts are now required to take advantage of recent technological advances to obtain high-resolution information that will enable us to build a spatial model of plant nuclear organisation, ideally at the nanoscale level and integrating all compartments. This ambitious task requires two steps: (i) a cultural change in the community, breaking the reticence to employ image processing and transcending qualitative analyses to quantitative analyses, and (ii) the active cooperation between life scientists, microscope providers and image analysts, including experts in deep learning approaches.

High-resolution imaging of specific nuclear probes and tagged components, aided by 3D quantitative image processing, have already provided insight into the different sub-nuclear compartments and their arrangement and composition at the sub-micrometric scale. We now need to transcend descriptive approaches and gain a biological understanding of these functional structures during plant growth, development, adaptation and responses to environmental variables (light, temperature, stress). Hence nuclear dynamics must be captured in an ‘integration-response’ context. This demands the development of approaches to follow the 3D nucleus over time and within the tissue context, while maintaining sufficient spatial resolution to resolve molecular components in action. We argue and envisage that the analysis of nuclear responses should be addressed by a combination of time-lapse imaging but also by reconstructions or pseudo-temporal sequences *in silico*, since each approach compensates for the deficit in the other.

We have recently seen considerable progress in time-lapse imaging of the nucleus *in vivo*, but spatial resolution remains seriously hampered by physical and optical problems posed by plant tissues and fluorescence viability under reported standard imaging conditions. Yet, thanks to enormous progress made in tissue clarification, probe labelling and increasingly sensitive microscopy imaging, the field is moving forward. In addition to classical spinning disk, confocal laser scanning and multiphoton-excitation microscopes, additional possibilities are offered for imaging nuclei *in depth* in plant tissues that merit exploration: photoacoustic tomography combining light absorption and acoustic detection [69] would allow tracking discrete states of the nucleus (*e.g*. using quantitative nuclear reporters) over time within millimetres of depth. Light sheet microscopy has been adopted in the plant science field in recent years [70] to offer another attractive option with high temporal resolution, although this is currently at the expense of spatial resolution and is limited by computationally expensive image processing.

Data analysis is another future challenge. We posit that a significant progress could be made with the customization of unbiased feature discovery and machine learning-based data analyses, such as those deployed in systems biology [162]. However, image analysis is not a simple task. This review does not intend to provide an exhaustive assessment of existing possibilities but rather to sketch some affordable solutions. The Bioimage Informatics Search Engine (BISE. http://biii.eu/) is associated with a forum enabling evaluation of an image analysis method for individual case studies. The emergence of automated pipelines for multi-angle image reconstruction [163] and machine-learning-based algorithms for reconstructing images at nanoscale resolution [140] promise tremendous progress in the coming decades. Moving from supervised learning to ‘intelligent’ image processing is also highly desirable for speeding up feature recognition and segmentation from complex 3D/4D images. Plant-specific datasets are needed to train the algorithms for plant nucleus-specific feature classification. Establishing a plant-specific nuclei image repository is therefore necessary and should be fostered by the community and consortium-based funding. Here, we made 3D images and 4D movies available through an OMERO repository (login ‘Public’, password ‘omero’) provided by the Florida State University Biological Science Dept. (omero.bio.fsu.edu) [164]. Allowing more researchers in plant sciences to store published datasets will allow for software/method benchmarking, as has been initiated through BIAFLOWS (https://www.biaflows.neubias.org; login: guest/password: guest).

*In silico* reconstruction of temporal sequences, although inherently only a proxy to reality, allows researchers to focus on large-scale measurements of the plant cell nucleus at high/super resolution. The idea is to probe and image hundreds of nuclei (isolated or in fixed/cleared tissues) from several plant tissue replicates at different time points of (ideally) ultra-controlled treatments. The development of semi-automated image acquisition and supervised-learning/batch image processing pipelines has begun and will require sustained efforts over coming years to improve availability and versatility. Overall, these prospects will require sustained collaborative efforts between biologists, biophysicists and computational scientists as well as dedicated research programs to enable synergies and platforms and cluster infrastructures for imaging, image processing and data analyses. Such initiatives are partially in place for other biological areas and are thus a matter for integrative efforts to drive additional synergies:- on the plant scientists’ side, to promote the great biological interest in plant cell nuclei, and on the established consortia’ side, to welcome additional model systems that offer unique biological and evolutionary insights into the complex command centres of plants with their amazing diversity and adaptation potential.
