# Supplementary material for: Probing the 3D architecture of the plant nucleus with microscopy approaches: challenges and solutions
Source: Nucleus. 2019 Jul 30;10(1):181–212. doi: 10.1080/19491034.2019.1644592 (PMC6682351; doi:10.1080/19491034.2019.1644592)
Supplement: Supplemental Material [file kncl-10-01-1644592-s005.docx]

SUPPLEMENTAL MATERIALS

**Supplemental File 1 raw data: Automatic tracking of nuclei**

**To be found at:** https://phaidra.univie.ac.at/o:925447 **Movie 5**

Maximum intensity projections of a 4.5 h time-lapse experiment conducted on the elongation zone of an Arabidopsis root expressing H2B-RFP, labelling chromatin. The microscope stage was readjusted to follow the same nuclei over time using the second version of TARDIS software. Time from the start of the experiment is indicated in h:min at the top left corner. Time points were acquired at 10 min intervals. Post-acquisition registration was made with the “Correct 3D drift” plugin in Fiji.

**Supplemental File 2 raw data: Semi-automatic tracking of nuclei I**

**To be found at:** <https://phaidra.univie.ac.at/o:925445> **Movie 3**

Maximum intensity projections of a 7 h time-lapse experiment conducted on the root tip of an Arabidopsis plant expressing H2A10-RFP, labelling chromatin. The microscope stage was readjusted to follow the same nuclei over time using the first version of TARDIS software. Time from the start of the experiment is indicated in h:min at the top left corner. Time points were acquired at 10 min intervals. Post-acquisition registration was made with the “Correct 3D drift” plugin in Fiji.

**Supplemental File 3 raw data**: **Live imaging of nuclei under heat stress**

**Tobe found at:** <https://phaidra.univie.ac.at/o:925451> **Movie 6**

Maximum intensity projections of a 30 h time-lapse experiment conducted on the root of an Arabidopsis plant expressing H2B-RFP in wt background during HS treatment. Time from the start of the experiment is indicated in h at the top left corner. Time points were acquired at 30 min intervals.

**Supplemental File 4 raw data**: **Live imaging of nuclei under ambient temperature (mock treatment)**

**To be found at:** <https://phaidra.univie.ac.at/o:925452> **Movie 7**

Maximum intensity projections of a 30 h time-lapse experiment conducted on the root of an Arabidopsis plant expressing H2B-RFP in wt background during mock treatment. Time from the start of the experiment is indicated in h at the top left corner. Time points were acquired at 30 min intervals.
